# Vaxign2: the second generation of the first Web-based vaccine design program using reverse vaccinology and machine learning

**DOI:** 10.1093/nar/gkab279

**Published:** 2021-05-01

**Authors:** Edison Ong, Michael F Cooke, Anthony Huffman, Zuoshuang Xiang, Mei U Wong, Haihe Wang, Meenakshi Seetharaman, Ninotchka Valdez, Yongqun He

**Affiliations:** Department of Computational Medicine and Bioinformatics, University of Michigan Medical School, Ann Arbor, MI 48109, USA; School of Information, University of Michigan, Ann Arbor, MI 48109, USA; Undergraduate Research Opportunity Program, College of Literature, Science, and the Arts, University of Michigan, Ann Arbor, MI 48109, USA; Department of Computational Medicine and Bioinformatics, University of Michigan Medical School, Ann Arbor, MI 48109, USA; Unit for Laboratory Animal Medicine, University of Michigan Medical School, Ann Arbor, MI 48109, USA; Unit for Laboratory Animal Medicine, University of Michigan Medical School, Ann Arbor, MI 48109, USA; Department of Pathogenobiology, Daqing Branch of Harbin Medical University, Daqing, Helongjiang, China; Undergraduate Research Opportunity Program, College of Literature, Science, and the Arts, University of Michigan, Ann Arbor, MI 48109, USA; Undergraduate Research Opportunity Program, College of Literature, Science, and the Arts, University of Michigan, Ann Arbor, MI 48109, USA; Department of Computational Medicine and Bioinformatics, University of Michigan Medical School, Ann Arbor, MI 48109, USA; Unit for Laboratory Animal Medicine, University of Michigan Medical School, Ann Arbor, MI 48109, USA; Department of Microbiology and Immunology, University of Michigan Medical School, Ann Arbor, MI 48109, USA

## Abstract

Vaccination is one of the most significant inventions in medicine. Reverse vaccinology (RV) is a state-of-the-art technique to predict vaccine candidates from pathogen's genome(s). To promote vaccine development, we updated Vaxign2, the first web-based vaccine design program using reverse vaccinology with machine learning. Vaxign2 is a comprehensive web server for rational vaccine design, consisting of predictive and computational workflow components. The predictive part includes the original Vaxign filtering-based method and a new machine learning-based method, Vaxign-ML. The benchmarking results using a validation dataset showed that Vaxign-ML had superior prediction performance compared to other RV tools. Besides the prediction component, Vaxign2 implemented various post-prediction analyses to significantly enhance users’ capability to refine the prediction results based on different vaccine design rationales and considerably reduce user time to analyze the Vaxign/Vaxign-ML prediction results. Users provide proteome sequences as input data, select candidates based on Vaxign outputs and Vaxign-ML scores, and perform post-prediction analysis. Vaxign2 also includes precomputed results from approximately 1 million proteins in 398 proteomes of 36 pathogens. As a demonstration, Vaxign2 was used to effectively analyse SARS-CoV-2, the coronavirus causing COVID-19. The comprehensive framework of Vaxign2 can support better and more rational vaccine design. Vaxign2 is publicly accessible at http://www.violinet.org/vaxign2.

## Graphical Abstract

**Graphical Abstract:**
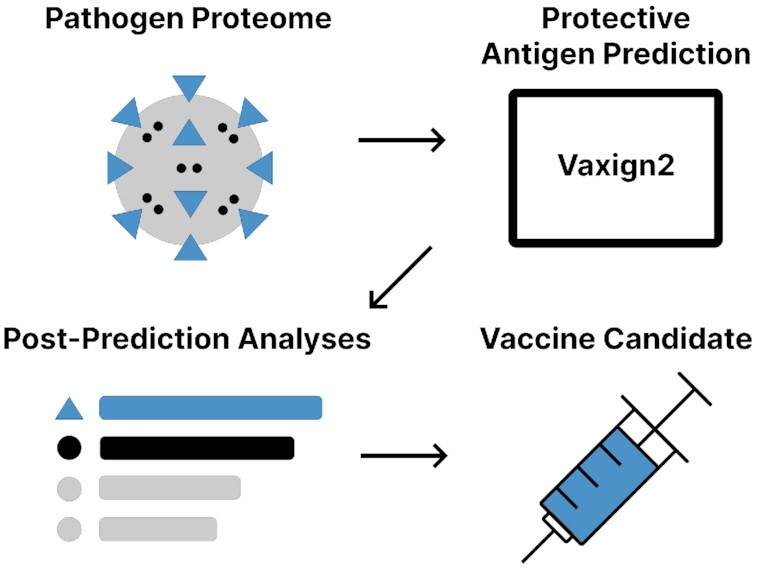
Vaxign2 is the first comprehensive web server application for vaccine design using reverse vaccinology and machine learning, showing superior prediction performance compared to other similar tools. Vaxign2 consists of (i) protective antigen prediction from a user-provided protein(s) or proteome of the target pathogen and (ii) post-prediction analyses of protein functions and immunogenicity.

## INTRODUCTION

Vaccination is one of the most significant inventions in the medical field, and WHO estimates about 2–3 million deaths are prevented through vaccination every year (1). Since Edward Jenner introduced a live attenuated vaccine against smallpox in 1798 (2), many different advanced vaccine types have been created, such as subunit, viral vector and nucleic acid vaccines. However, the first and the most crucial step of the development of all these advanced vaccine types is to select one or more protective antigens (PAgs), which could be a gene encoding a protein or the protein itself. The conventional approach has been time-consuming, but in 2000, the revolutionary technique of Reverse Vaccinology (RV) emerged, dramatically reducing the time required to identify PAgs from 5–15 years to 1–2 years (3,4). This success has led to the creation of various RV tools. Current open-source RV tools can be grouped into two categories, using filtering-based or machine learning (ML)-based methods. The filtering-based tools include Vaxign, the first web-based RV tool (5), and other tools such as NERVE (6), Jenner-predict server (7) and VacSol (8). The second type of RV tool leverages the power of ML to predict PAgs, including VaxiJen (9), Bowman's method (10) and Heinson's method (11). As reviewed by Dalsass *et al.*, the best model at that time achieved a recall of 0.76, and many of these tools lack a user-friendly interface for experimental scientists and standalone software for bioinformatics users (12).

As mentioned previously, we published the first web-based RV tool Vaxign in 2010 (5), and the original Vaxign manuscript is well-cited in the field of vaccine design and immunoinformatics. The Vaxign web service has been running since 2010 and is accessed by thousands of users per year. Over the past decade, Vaxign has been applied by other research groups to predict vaccine candidates against different pathogens such as *Helicobacter pylori* (13), *Mycobacterium tuberculosis* (14), and African swine fever virus (15). To push the performance of ML-based RV prediction further, we created the ML-based Vaxign, or Vaxign-ML, in 2020. A significant advantage of Vaxign-ML was that the training data to build the ML model was retrieved from the Protegen database, which stored over ten years of experimentally verified protective antigens from published literature. As a result, Vaxign-ML showed superior predictive performance compared to existing RV tools. The initial version of Vaxign-ML primarily focused on bacterial protective antigen prediction and was extended to predict viruses and parasites in the following updates. Then, Vaxign-ML was applied to predict COVID-19 vaccine candidates, with the SARS-CoV-2 spike (S) glycoprotein being the top candidate followed by the non-structural protein 3 (nsp3). The S protein is the primary target of most COVID-19 vaccines, including the Pfizer (16) and Moderna (17) mRNA vaccines with high reported efficacy in Phase 3 clinical trials. The second candidate predicted by Vaxign-ML, nsp3 protein, contained the Papain-Like protease (PLpro) sub-domain, which was reported to play a critical role in the SARS-CoV-2 evasion mechanism against host antiviral immune responses (18). The inhibition of PLpro impaired the virus-induced cytopathogenic effect and reduced viral replication in infected cells.

Here, we present the Vaxign2 web server, a comprehensive tool to facilitate rational vaccine design. Vaxign2 consists of a predictive framework and a computational workflow component. The predictive framework includes the original Vaxign filtering-based method and the newly developed Vaxign-ML machine learning-based method. Vaxign2 also implemented an array of post-prediction analyses besides the prediction framework, including epitope prediction, population coverage, and functional analysis. These analyses significantly enhance user capability to refine the prediction results based on different vaccine design rationales and access the biological function and immunogenic content of Vaxign and Vaxign-ML prediction results.

## METHODS AND IMPLEMENTATION

**Figure 1.**
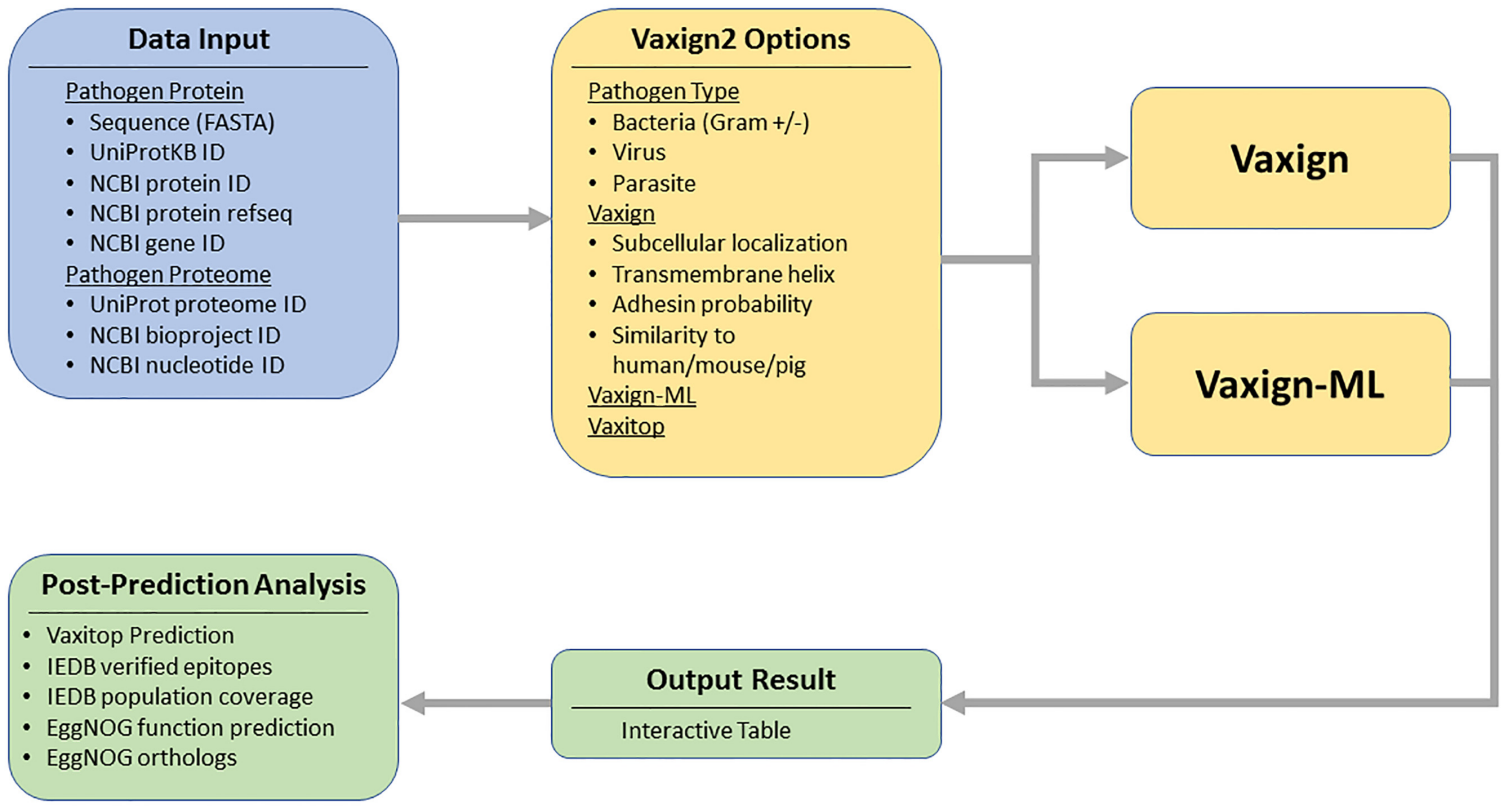
The overall workflow of Vaxign2. Users provide the input data in the form of pathogen protein or proteome (blue box). Then the users can select Vaxign2 options in the web interface and submit the prediction query (yellow boxes). A Vaxign2 summary page will display the Vaxign-ML scores, and users can perform post-prediction analysis on the selected protein (green boxes).

The input of Vaxign2 is the pathogen protein or proteome sequences (Figure 1). For protein sequences, users can predict PAgs by directly inputting the amino acid sequences in FASTA format or providing one of the following identifiers: UniProtKB ID, NCBI protein ID, NCBI protein RefSeq or NCBI gene ID. Vaxign2 also supports retrieval of the entire proteome amino acid sequences from the corresponding database identifiers, including UniProt proteome ID, NCBI bioproject ID or NCBI nucleotide ID, to perform PAg prediction for the entire pathogen proteome. Users then select options in the web interface and submit the prediction query. Once all processes are finished, a Vaxign2 summary page will display the generated Vaxign-ML scores and Vaxign predicted biological properties. By default, the result is ranked based on the Vaxign-ML score (recommended threshold = 90.0), which is the percentile rank score from the Vaxign-ML prediction. Vaxign2 also inherits the original Vaxign filtering-based method. It allows users to select output protein based on subcellular localization, the number of transmembrane domains, adhesin probability, and similarity to host proteins (human/mouse/pig) if enabled during Vaxign2 option selection. Finally, users can select individual protein from the summary page for further post-prediction analyses, including Vaxitop epitope prediction, verified epitope mapping, epitope population coverage prediction, protein function prediction and protein ortholog identification.

### Vaxign and Vaxign-ML predictive framework

#### Vaxign filtering-based protective antigen prediction

Vaxign is the first web-based vaccine design program using RV. The first generation of Vaxign applies a filtering-based method to select vaccine antigen candidates based on the user's prior knowledge of the target pathogen's pathogenesis. A typical workflow involves the following components: (i) subcellular localization computed by PSORTb program (19); (ii) transmembrane domains computed using TMHMM 2.0 with default settings (20); (iii) adhesin probability calculated using SPAAN program with default settings (21); (iv) similarity to host proteins (human/mouse/pig) using BLAST and NCBI protein database (22).

#### Vaxign-ML machine learning-based protective antigen prediction

With the advance of machine learning and accumulation of manually collected protective antigens in Protegen (23), Vaxign-ML was created and significantly improved vaccine antigen prediction (24). In brief, Vaxign-ML combined the protein sequences’ biological and physicochemical properties as the input features to train five different machine learning models. The input protein sequences were extracted from the Protegen database, a continuous effort over the past ten years collecting and annotating experimentally verified protective antigens (23). All machine learning models were evaluated and selected based on nested five-fold cross-validation and leave-one-pathogen-out validation. The best performing model, extreme gradient boosting, was used to build the Vaxign-ML program.

### Vaxign2 post-prediction analysis workflow

#### Vaxitop epitope prediction and IEDB verified epitope mapping

However, the Vaxign and Vaxign-ML predicted PAgs could be further investigated for their immunogenic potential as vaccine candidates before experimental verification. Vaxign2 provides the immunogenicity assessment by the post-prediction analysis workflow. The principal mechanism of vaccines is the adaptive immune response: humoral (antibody) and cell-mediated responses. The protection offered by these immune responses is primarily mediated by B cells and T cells. In particular, T cell response can be mainly categorized into CD4 (helper) and CD8 (cytotoxic) T cell responses, which are induced by epitopes bound to major histocompatibility complex (MHC)-II, and MHC-I molecules, respectively. Therefore, it is essential to evaluate the predicted PAgs based on their B cell and T cell epitopes.

Vaxign2 supports MHC-I and MHC-II T cell epitope predictions for input proteins via Vaxitop. In brief, all the epitopes’ Position-Specific Scoring Matrix (PSSM) for different MHC-I or MHC-II alleles are generated by MEME (25) based on known epitope data from the IEDB immune epitope database (26). Then the input proteins are scanned for epitopes by the PSSMs. The *P*-value for the predicted epitope binding to PSSMs is calculated by the MAST sequence homology search algorithm (25). Besides epitope prediction, Vaxign2 also supports the mapping of IEDB experimentally verified T cell and B cell epitopes to the input proteins (26).

#### Population coverage prediction

As mentioned in the previous section, epitopes bound to the MHC-I or MHC-II molecules are presented to T cells to induce an immune response. However, human MHC molecules are highly polymorphic, and the expression of different MHC molecules is significantly impacted by human genetic variation. Thus, it is essential to determine if the predicted PAg contains a set of epitopes capable of binding to different MHC molecules and offers a broad coverage to the world population. Based on the result from Vaxitop MHC-I and MHC-II T cell epitope prediction, Vaxign2 can also calculate the population coverage of the input proteins using the IEDB Population Coverage Tool (27). The predicted population coverage of the different countries is also visualized and highlighted in the world map.

#### Protein function and orthologs prediction

The sequences of all PAgs are scanned for functional domains, including Clusters of Orthologs (COG) and Gene Ontology (GO) terms, as well as possible orthologous proteins using HMMER2 (http://hmmer.org/) with the hidden Markov models downloaded from the EggNog database (28).

## RESULTS

### Vaxign and Vaxign-ML benchmarking

A benchmarking dataset was created to evaluate Vaxign and Vaxign-ML to other existing open-source RV tools, including VaxiJen3 (9) and Antigenic (29). This benchmarking dataset was composed of two external resources: (i) Dalsass *et al.*: 100 positive samples (12); (ii) Heinson *et al.*: 200 positives and 200 negatives (11). To avoid biased evaluation and over-fitting, all samples were aligned to the Vaxign-ML training data, and all identical or similar protein sequences were removed from the benchmarking dataset. The 200 negatives were also checked to ensure that no experimental evidence was reported in the literature. The final benchmarking dataset consisted of 131 positives and 118 negatives. The benchmarking result showed that Vaxign had the highest precision with the cost of the lowest recall (Table 1). Overall, Vaxign-ML had the highest recall, weighted F1- score, and Matthew's correlation coefficient compared to other RV tools.

**Table 1.** Benchmarking performance of Vaxign and Vaxign-ML comparing to other open-source reverse vaccinology tools

Tools	Recall	Precision	WF1	MCC
**Vaxign-ML**	0.81	0.75	0.76	0.51
**Vaxign**	0.32	0.79	0.56	0.27
**VaxiJen3**	0.78	0.71	0.71	0.42
**Antigenic**	0.5	0.52	0.49	-0.02

Abbreviation: WF1 = weighted F1 score. MCC = Matthew's correlation coefficient.

### Vaxign2 Pre-computed queries

Vaxign2 contains publicly available pre-computed results of 980,285 proteins from 398 proteomes in 36 pathogens (Supplementary Table S1), and Table 2 listed 13 pathogens with at least ten proteomes analyzed. Compared to the original Vaxign, Vaxign2 added 19, 322 and 789 093 new pathogens, proteomes and proteins to the pre-computed queries, respectively. In addition, Vaxign2 also incorporated the Vaxign-ML predictions into the pre-computed query pipeline. Compared to the original Vaxign, New post-analysis features such as epitope population coverage and ortholog phylogeny generation were also added.

**Table 2.** Vaxign2 pre-computed queries with at least 10 proteomes. Full list can be found in Supplemntal Table S1

Pathogen name	# of Proteome	# of proteins
*Streptococcus*	53	105 632
*Herpesvirus*	52	5104
*Acinetobacter baumannii*	35	131 070
*Staphylococcus aureus*	33	86 662
*Brucella*	31	98 888
*Salmonella*	23	104 009
*Vibrio*	22	50 267
*Mycobacterium*	15	64 073
*Corynebacterium*	14	33 665
*Clostridium difficile*	13	48 849
*Escherichia coli*	11	53 932
*Campylobacter*	10	17 445
*Clostridium*	10	35 130
**Total**	**398**	**980 285**

Vaxign, Vaxign-ML and Vaxign2 have been used in many studies in vaccine design, pathogenesis mechanism studies, and genome analysis. The Vaxign and Vaxign-ML predictive framework has been applied to predict PAgs for vaccine development against over 20 pathogens (Supplementary Table S2). In many studies, researchers applied Vaxign and Vaxign-ML to predict vaccine antigen targets, but Vaxign was also used to study the virulence of *Clostridioides difficile* cell wall protein 22 (Cwp22) (30) and to select vaccine targets for antibiotic-resistant *Acinetobacter baumannii* (31).

### Use case study

The emerging Coronavirus Disease 2019 (COVID-19) pandemic poses a massive crisis to global public health, and WHO declared the COVID-19 as a pandemic on 11 March 2020. The causative agent of COVID-19 is SARS-CoV-2, which shares high sequence identity with SARS-CoV (32). As of 6 February 2021, this on-going COVID-19 pandemic had caused over 105 million infection cases and over 2.3 million deaths globally. To effectively control the spread of this deadly virus, it is important to develop safe and effective COVID-19 vaccines.

#### Use Case 1: dynamic analysis of SARS-CoV-2 S protein evaluation

**Figure 2.**
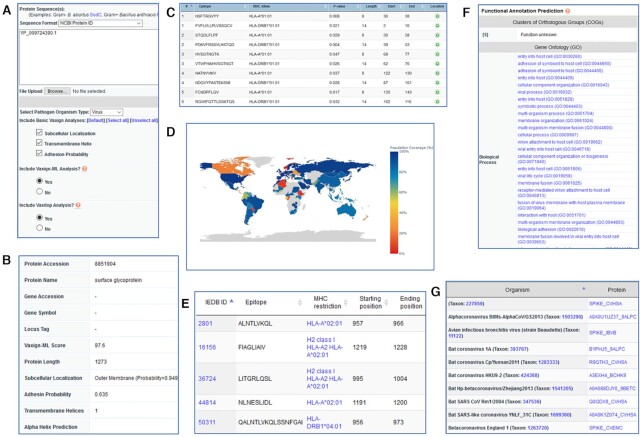
Dynamic analysis of SARS-CoV-2 S protein in Vaxign2. (**A**) The protein accession number of S protein was used as the input, together with the selection of specified parameters. (**B**) The basic analysis results were provided for the S protein. (**C**) Vaxitop predicted human MHC-I & -II epitopes and users could select the result based on different MHC Classes, MHC Alleles and epitope length. (**D**) Population coverage of S protein's predicted epitopes was computed using the MHC-I & -II reference alleles for the general population of each country. Note that some countries with low predicted population coverage might not reflect the actual population coverage due to the lack of reported allele frequencies in the Allele Frequency Net Database (36). (**E**) Vaxign2 searched the IEDB Epitope database to provide a list of experimentally verified epitopes for both B and T cells. (**F**, **G**) EGGNOG was used as a database to identify matching functions, Gene Ontology terms, and known orthologs to facilitate rational vaccine antigen selection.

The SARS-CoV-2 S protein is a commonly used vaccine antigen in current COVID-19 vaccine development. Figure 2 showed how Vaxign2 was used to dynamically assess the S protein as a vaccine target by Vaxign/Vaxign-ML, and to evaluate the immunogenicity and biological functions of S protein in post-prediction analyses. The SARS-CoV-2 S protein's NCBI protein ID (YP_009724390.1) was input to the Vaxign2 dynamic analysis (Figure 2A). Vaxign2 computed Vaxign/Vaxign-ML results, including the Vaxign-ML score and adhesin probability. Vaxign-ML predicted S protein to be a good vaccine antigen with a score of 97.6 (Figure 2B). Vaxign calculated S protein's adhesion probability of 0.635; with the cutoff of 0.51, this protein was protected to be an adhesin contributing to viral entry into the host cell. The Vaxign/Vaxign-ML results both suggested S protein as a promising vaccine antigen target.

The S protein was then evaluated for its immunogenicity and functional profile by Vaxign2 post-prediction analyses. Vaxitop predicted 94 MHC-I (Supplementary Table S3) and 54 MHC-II (Supplementary Table S4) unique promiscuous epitopes for S protein (*P*-value ≤ 0.01) (Figure 2C). The MHC-I & -II reference alleles represent the majority of human MHC alleles in the world population (33,34), and epitope promiscuity is defined to bind four or more MHC-I or MHC-II alleles in the reference set (35). Vaxign2 also found 12 and 45 verified epitopes for T and B cells, respectively (Figure 2D, Supplementary Tables S5 and S6). Furthermore, S protein was predicted to have high population coverage in most countries (Figure 2E). Note that some countries with low or no predicted population coverage might be due to the lack of reported allele frequencies in the Allele Frequency Net Database (36) and did not reflect the actual population coverage. Vaxign2 also computed the Gene Ontology (GO) terms for S protein and identified virulence-related terms (Figure 2F), such as viral entry into host cell (GO:0046718), host cell surface receptor binding (GO:0046789), and receptor-mediated virion attachment to host cell (GO:0046813) (Supplementary Table S7). Finally, a total of 51 S protein orthologs were identified (Figure 2G, Supplementary Table S8) in *Orthocoronavirinae*, which is a subfamily related to human coronaviruses. In summary, the Vaxign2 post-prediction analyses suggested S protein had good epitope profiles and contributed to an important role in viral infection. Such analyses provided by Vaxign2 provided valuable biological rationales on the selection of S protein as a vaccine candidate. Indeed, S protein has been the primary target of many COVID-19 vaccines such as Pfizer and Moderna (16,17).

#### Use Case 2: pre-computed queries for coronaviruses vaccine selection

**Figure 3.**
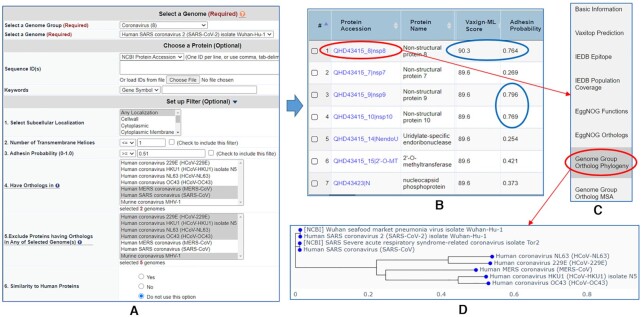
Comparison of multiple coronavirus strains for uniquely conserved strains. (**A**) Query for SARS-CoV-2 proteins that share orthologs in SARS-CoV and MERS-CoV but not in four other human coronaviruses and one murine coronavirus strain. (**B**) The results of seven proteins including nsp8 predicted as a protective antigen and three proteins (nsp8–10) as adhesin proteins. (**C**) Selection of nsp8 for further analysis. (**D**) The result of nsp8’s genome group ortholog phylogeny.

The complete proteome of SARS-CoV-2 was uploaded to the Vaxign2 pre-computed queries and was compared to seven other coronaviruses (Figure 3). The causative agents for the Middle East respiratory syndrome (MERS) and Severe acute respiratory syndrome (SARS) are MERS-CoV and SARS-CoV, respectively. SARS-CoV, SARS-CoV-2, and MERS-CoV are all beta-coronaviruses, which are very virulent and cause severe respiratory syndromes. On the other hand, human coronavirus OC43 (HCoV-OC43) and HKU1 (HCoV-HKU1) belong to the beta-coronavirus, while human coronavirus 229E (HCoV-229E) and NL63 (HCoV-NL63) are alpha-coronaviruses. These four strains only cause mild cold symptoms in humans. In addition to the human coronaviruses mentioned above, a murine coronavirus MHV-1 was also included in the comparison to SARS-CoV-2. The hypothesis is that some coronavirus virulence factors only exist in the severe form of SARS-CoV/SARS-CoV-2/MERS-CoV but not in the other mild or non-human coronaviruses. The pre-computed coronavirus results in Vaxign2 could be queried (Figure 3A) to address this hypothesis. Specifically, our Vaxign2 query found seven proteins that were conserved in the three virulent human coronaviruses (SARS-CoV, SARS-CoV-2 and MERS-CoV), but not in the other five mild or non-human coronaviruses. These seven proteins included Non-structural protein 7–10 (nsp7–10), Uridylate-specific endoribonuclease (nendoU), 2′-*O*-methyltransferase (2′-*O*-MT), and nucleocapsid phosphoprotein (N) (Figure 3B). Among the seven conserved proteins, three proteins (nsp8–10) were predicted as adhesion proteins by Vaxign, but only nsp8 protein was predicted to be PAg by Vaxign-ML. Therefore, nsp8 was selected for further analysis (Figure 3C). In particular, the genome group phylogeny analysis (Figure 3D) showed that nsp8 was predicted to be more closely related to the SARS-CoV than MERS-CoV and the other four mild human coronaviruses (Figure 3D). It could be a feasible strategy to create a COVID-19 cocktail vaccine, as described in our COVID-19 vaccine prediction study (37), that combines multiple proteins to target different aspects of host immunity for better protection.

## CONCLUSION AND FUTURE DIRECTION

Vaxign2 is a comprehensive system providing protective antigen (PAg) prediction and post-prediction analysis to support accurate and efficient antigen selection during the early step of vaccine development. The original Vaxign is one of the most popular open-source Reverse Vaccinology (RV) tools. Vaxign-ML is a machine learning (ML)-based RV prediction tool that facilitates vaccine candidate selection with high accuracy. The current Vaxign-ML was primariy developed for bacterial and viral PAg prediction, and will be extended to predict parasitic PAgs. By integrating Vaxign and Vaxign-ML, Vaxign2 provides an accurate PAg predict and yet supports customizable selection based on the user's prior knowledge. Furthermore, Vaxign2 facilitates post-prediction analysis of the predicted PAgs for immunogenicity and functional assessments.

Vaccine informatics (38) is a rapidly developing field, and many new technologies could be integrated into the Vaxign2 system to not only improve the antigen selection process but also support antigen optimization. First, with the accumulation of PAgs in the literature, it is feasible to apply deep learning to improve the RV-based antigen selection process further. The type of immune responses (e.g. Th1 and Th2 responses) induced by these PAgs and post-translation modification (e.g., glycosylation sites) could also be mined from the literature and enhance Vaxign2 predictions. Second, Structural Vaccinology (SV) is an emerging field to rationally design vaccine antigens and has been applied to the respiratory syncytial virus (39) and SARS-CoV-2 (40). Integration of Vaxign2 and SV can promote antigen selection and optimization. The continuous development of Vaxign2 presents the best opportunity for the rapid development of effective and safe vaccines.

## DATA AVAILABILITY

Vaxign2 is accessible at http://www.violinet.org/vaxign2. The Vaxign2 source code is also available in the GitHub repository (https://github.com/VIOLINet/Vaxign2-django).

## Supplementary Material

gkab279_Supplemental_File

## Notes

Present address: Edison Ong, GlaxoSmithKline, Rixensart 1330, Belgium.
