# Supplementary material for: Vaxign2: the second generation of the first Web-based vaccine design program using reverse vaccinology and machine learning
Source: Nucleic Acids Res. 2021 May 1;49(W1):W671–8. doi: 10.1093/nar/gkab279 (PMC8218197; doi:10.1093/nar/gkab279)
Supplement: gkab279_Supplemental_File [file gkab279_supplemental_file.pdf]

Table S1. Vaxign2 pre-computed queries.

Pathogen Name	No. of Strain (or RefSeq Accession)	No. of proteins
Streptococcus	53	105632
Herpesvirus	52	5104
Acinetobacter baumannii	35	131070
Staphylococcus aureus	33	86662
Brucella	31	98888
Salmonella	23	104009
Vibrio	22	50267
Mycobacterium	15	64073
Corynebacterium	14	33665
Clostridium difficile	13	48849
Escherichia coli	11	53932
Campylobacter	10	17445
Clostridium	10	35130
Francisella	9	14772
Legionella pneumophila	9	28118
Coronavirus	8	171
Helicobacter pylori	6	9261
Coxiella burnetii	5	9686
Shigella	5	21517
Haemophilus influenzae	4	6735
Lymphocystis virus	4	515
Mycoplasma mycoides	4	3773
Neisseria meningitidis	4	7909
Bacillus anthracis	3	16182
Listeria monocytogenes	3	8641
Mycoplasma hyopneumoniae	3	2005
Aeromonas hydrophila	2	8555
Human immunodeficiency virus	2	33
Mycoplasma bovis	2	1566
Flavobacterium columnare	1	2642
Measles virus	1	7
Rickettsia	1	835
Streptococcus pneumoniae	1	2202
Vaccinia virus	1	223
Variola virus	1	197
	1	14
**Total**	**398**	**980285**

Table S2. Vaxign/Vaxign-ML/Vaxign2 citations.

Pub. Year	Disease	Pathogen	Title	Journal	PMID	PMCID	DOI
2020	Urinary tract infection	*P. mirabilis*, and *K. pneumonia* strains	Protective multi-epitope candidate vaccine for urinary tract infection	*Biotechnology Reports*	33304840	PMC7711219	10.1016/j.btre.2020.e00564
2020	COVID-19	SARS-CoV-2	COVID-19 coronavirus vaccine design using reverse vaccinology and machine learning	*Frontiers in Immunology*	32719684	PMC7350702	10.3389/fimmu.2020.01581
2020		*Staphylococcus aureus*	Design of Staphylococcus aureus New Vaccine Candidates with B and T Cell Epitope Mapping, Reverse Vaccinology, and Immunoinformatics	*A Journal of Integrative Biology*	32286190		10.1089/omi.2019.0183
2020		*A. baumannii*	New putative vaccine candidates against Acinetobacter baumannii using the reverse vaccinology method	*Microbial Pathogenesis*	32145321		10.1016/j.micpath.2020.104114
2020		*A. baumannii*	Analysis of the Extracellular Proteome of Colistin-Resistant Korean Acinetobacter baumannii Strains	*ACS Omega*	32226849	PMC7097930	10.1021/acsomega.9b03723
2020	Shigellosis	*Shigella spp.*	Reverse vaccinology approach for the identification and characterization of outer membrane proteins of Shigella flexneri as potential cellular- and antibody-dependent vaccine candidates	*Clinical and Experimental Vaccine Research*	32095437	PMC7024733	10.7774/cevr.2020.9.1.15
2019		*Bacteroides fragilis*	Designing a multi-epitopic vaccine against the enterotoxigenic Bacteroides fragilis based on immunoinformatics approach	Scientific Reports	31874963	PMC6930219	10.1038/s41598-019-55613-w
2019	Pneumonia	*Mycoplasma pneumoniae*	Reverse vaccinology and subtractive genomics reveal new therapeutic targets against Mycoplasma pneumoniae: a causative agent of pneumonia	*Royal Society Open Science*	31417766	PMC6689572	10.1098/rsos.190907
2019	C. diff infection	*C. difficile*	Cwp22, a novel peptidoglycan cross-linking enzyme, plays pleiotropic roles in Clostridioides difficile	*Environ Microbiology*	31173438	PMC7219524	10.1111/1462-2920.14706
2018			Identification of Cross-Protective Potential Antigens against Pathogenic Brucella spp. through Combining Pan-Genome Analysis with Reverse Vaccinology	*Journal of Immunology Research*	30622973	PMC6304850	10.1155/2018/1474517
2018		*Streptococcus pneumoniae*	Novel Immunoprotective Proteins of Streptococcus pneumoniae Identified by Opsonophagocytosis Killing Screen	*Infection and Immunity*	29891544	PMC6105882	10.1128/IAI.00423-18
2018		*H. pylori*	Prediction of Epitopes in the Proteome of Helicobacter pylori	*Global Journal of Health Science*			10.5539/gjhs.v10n7p148
2018	Chancroid	*Haemophilus ducreyi*	Putative vaccine candidates and drug targets identified by reverse vaccinology and subtractive genomics approaches to control Haemophilus ducreyi, the causative agent of chancroid	*Journal of the Royal Society Interface*	29792307	PMC6000166	10.1098/rsif.2018.0032
2018		*Vibrio anguillarum*	Potential Outer Membrane Protein Candidates for Vaccine Development Against the Pathogen Vibrio anguillarum: A Reverse Vaccinology Based Identification	*Current Microbiology*	29119233		10.1007/s00284-017-1390-z
2017		*Acinetobacter baumannii*	Immunoprotective potential of BamA, the outer membrane protein assembly factor, against MDR Acinetobacter baumannii	*Scientific Reports*	28963492	PMC5622086	10.1038/s41598-017-12789-3
2017		*Burkholderia pseudomallei*	Use of Reverse Vaccinology in the Design and Construction of Nanoglycoconjugate Vaccines against Burkholderia pseudomallei	*Clinical and Vaccine Immunology*	28903988	PMC5674190	10.1128/CVI.00206-17
2017		*Mycobacterium spp.*	Computational Identification and Characterization of a Promiscuous T-Cell Epitope on the Extracellular Protein 85B of Mycobacterium spp. for Peptide-Based Subunit Vaccine Design	*BioMed Research International*	28401156	PMC5376426	10.1155/2017/4826030
2017		*E. coli*	Ontology-based literature mining of E. coli vaccine-associated gene interaction networks	*Journal of Biomedical Semantics*	28288685	PMC5348867	10.1186/s13326-017-0122-4

2017	African swine fever	African swine fever virus	Safety and immunogenicity of mammalian cell derived and Modified Vaccinia Ankara vectored African swine fever subunit antigens in swine	*Veterinary Immunology and Immunopathology*	28241999	PMC7112906	10.1016/j.vetimm.2017.01.004
2017		*Acinetobacter baumannii*	Antibiotic Resistance Determinant-Focused Acinetobacter baumannii Vaccine Designed Using Reverse Vaccinology	*International Journal of Molecular Sciences*	28230771	PMC5343991	10.3390/ijms18020458
2017	Syphilis	*Treponema pallidum*	An In Silico Identification of Common Putative Vaccine Candidates against Treponema pallidum: A Reverse Vaccinology and Subtractive Genomics Based Approach	*International Journal of Molecular Sciences*	28216574	PMC5343936	10.3390/ijms18020402
2017		*Pajaroellobacter abortibovis*	Genome Report: Identification and Validation of Antigenic Proteins from Pajaroellobacter abortibovis Using De Novo Genome Sequence Assembly and Reverse Vaccinology	*G3: Genes, Genomes, Genetics*	28040777	PMC5295582	10.1534/g3.116.036673
2016	Tuberculosis	*Mycobacterium tuberculosis*	In silico identification and characterization of a hypothetical protein of Mycobacterium tuberculosis EAI5 as a potential virulent factor	Bioinformation	28149053	PMC5267962	10.6026/97320630012182
2016	Campylobacteriosis	*Campylobacter spp.*	Identification of Novel Vaccine Candidates against Campylobacter through Reverse Vaccinology	*Journal of Immunology Research*	27413761	PMC4928009	10.1155/2016/5715790
2016		*A. baumannii*	Immunoprotective potential of in silico predicted Acinetobacter baumannii outer membrane nuclease, NucAb	*International Journal of Medical Microbiology*	26614015	PMC4751259	0.1016/j.ijmm.2015.10.005
		Brucella melitensis, *Brucella ovis*	Proteomic analysis of Brucella melitensis and Brucella ovis for identification of virulence factor using bioinformatics approachs	*Molecular and Cellular Probes*	32428653		10.1016/j.mcp.2020.101581
2015		*Corynebacterium urealyticum*	Genome informatics and vaccine targets in Corynebacterium urealyticum using two whole genomes, comparative genomics, and reverse vaccinology	*BMC Genomics*	26041051	PMC4460590	
2013		*Legionella pneumophila*	Genome Sequence of an Environmental Isolate of the Bacterial Pathogen Legionella pneumophila	*Genome Announcements / Microbiology Resource Announcements*	23792742	PMC3675512	10.1128/genomeA.00320-13
2013		*Brucella melitensis*	Immunogenic and invasive properties of Brucella melitensis 16M outer membrane protein vaccine candidates identified via a reverse vaccinology approach	*PLoS One*	23533646	PMC3606113	10.1371/journal.pone.0059751
2013		HSV1, HSV-2	Genome-wide prediction of vaccine targets for human herpes simplex viruses using Vaxign reverse vaccinology	*BMC Bioinformatics*	23514126	PMC3599071	10.1186/1471-2105-14-S4-S2
2012	Urinary track infection	*E. coli*	Preventing urinary tract infection: progress toward an effective Escherichia coli vaccine	*Expert Review of Vaccines*	22873125	PMC3498450	10.1586/erv.12.36
2010	Brucellosis	*Brucella spp.*	Bioinformatics analysis of Brucella vaccines and vaccine targets using VIOLIN	*Immunome Research*	20875156	PMC2946783	10.1186/1745-7580-6-S1-S5

Table S3. Vaxitop predicted MHC-I epitopes for SARS-CoV-2 S protein.

#	Epitope	MHC Allele	P-value	Length	Start	End
1	SQCVNLTTR	HLA-A*31:01	0.003	8	13	21
1	SQCVNLTTR	HLA-A*31:01	0.003	8	13	21
2	TTRTQLPPA	HLA-A*30:01	0.001	8	19	27
2	TTRTQLPPA	HLA-A*30:01	0.001	8	19	27
3	YTNSFTRGV	HLA-A*02:01	0.01	8	28	36
3	YTNSFTRGV	HLA-A*02:03	0.008	8	28	36
3	YTNSFTRGV	HLA-A*68:02	0.003	8	28	36
3	YTNSFTRGV	HLA-A*02:01	0.01	8	28	36
3	YTNSFTRGV	HLA-A*02:03	0.008	8	28	36
3	YTNSFTRGV	HLA-A*68:02	0.003	8	28	36
4	NSFTRGVYY	HLA-A*01:01	0.008	8	30	38
4	NSFTRGVYY	HLA-A*01:01	0.008	8	30	38
5	GVYYPDKVF	HLA-A*32:01	0.008	8	35	43
5	GVYYPDKVF	HLA-A*32:01	0.008	8	35	43
6	KVFRSSVLH	HLA-A*03:01	0.008	8	41	49
6	KVFRSSVLH	HLA-A*03:01	0.008	8	41	49
7	STQDLFLPF	HLA-B*15:01	0.003	8	50	58
7	STQDLFLPF	HLA-A*32:01	0.003	8	50	58
7	STQDLFLPF	HLA-A*26:01	0.001	8	50	58
7	STQDLFLPF	HLA-B*15:01	0.003	8	50	58
7	STQDLFLPF	HLA-A*32:01	0.003	8	50	58
7	STQDLFLPF	HLA-A*26:01	0.001	8	50	58
8	LPFFSNVTW	HLA-B*53:01	0.007	8	56	64
8	LPFFSNVTW	HLA-B*53:01	0.007	8	56	64
9	HVSGTNGTK	HLA-A*68:01	0.003	8	69	77
9	HVSGTNGTK	HLA-A*68:01	0.003	8	69	77
10	GTKRFDNPV	HLA-A*02:03	0.009	8	75	83
10	GTKRFDNPV	HLA-A*02:03	0.009	8	75	83
11	RFDNPVLPF	HLA-A*24:02	0.009	8	78	86
11	RFDNPVLPF	HLA-B*58:01	0.008	8	78	86
11	RFDNPVLPF	HLA-A*24:02	0.009	8	78	86
11	RFDNPVLPF	HLA-B*58:01	0.008	8	78	86
12	VLPFNDGVY	HLA-A*30:02	0.007	8	83	91
12	VLPFNDGVY	HLA-A*30:02	0.007	8	83	91
13	LPFNDGVYF	HLA-B*35:01	0.004	8	84	92
13	LPFNDGVYF	HLA-B*07:02	0.003	8	84	92
13	LPFNDGVYF	HLA-B*53:01	0.001	8	84	92
13	LPFNDGVYF	HLA-B*35:01	0.004	8	84	92
13	LPFNDGVYF	HLA-B*07:02	0.003	8	84	92
13	LPFNDGVYF	HLA-B*53:01	0.001	8	84	92
14	GVYFASTEK	HLA-A*03:01	0.001	8	89	97
14	GVYFASTEK	HLA-A*11:01	0.001	8	89	97
14	GVYFASTEK	HLA-A*31:01	0.003	8	89	97
14	GVYFASTEK	HLA-A*03:01	0.001	8	89	97
14	GVYFASTEK	HLA-A*11:01	0.001	8	89	97
14	GVYFASTEK	HLA-A*31:01	0.003	8	89	97
15	FASTEKSNI	HLA-B*51:01	0.002	8	92	100

15	FASTEKSNI	HLA-B*51:01	0.002	8	92	100
16	TEKSNIIRG	HLA-B*44:02	0.01	8	95	103
16	TEKSNIIRG	HLA-B*44:02	0.01	8	95	103
17	TLDSKTQSL	HLA-A*02:01	0.006	8	109	117
17	TLDSKTQSL	HLA-B*08:01	0.009	8	109	117
17	TLDSKTQSL	HLA-A*02:01	0.006	8	109	117
17	TLDSKTQSL	HLA-B*08:01	0.009	8	109	117
18	FQFCNDPFL	HLA-B*40:01	0.009	8	133	141
18	FQFCNDPFL	HLA-A*02:01	0.005	8	133	141
18	FQFCNDPFL	HLA-A*02:06	0.002	8	133	141
18	FQFCNDPFL	HLA-B*15:01	0.008	8	133	141
18	FQFCNDPFL	HLA-B*40:01	0.009	8	133	141
18	FQFCNDPFL	HLA-A*02:01	0.005	8	133	141
18	FQFCNDPFL	HLA-A*02:06	0.002	8	133	141
18	FQFCNDPFL	HLA-B*15:01	0.008	8	133	141
19	FCNDPFLGV	HLA-A*68:02	0.001	8	135	143
19	FCNDPFLGV	HLA-A*68:02	0.001	8	135	143
20	CNDPFLGVY	HLA-A*30:02	0.002	8	136	144
20	CNDPFLGVY	HLA-A*30:02	0.002	8	136	144
21	YYHKNNKSW	HLA-A*24:02	0.009	8	144	152
21	YYHKNNKSW	HLA-A*24:02	0.009	8	144	152
22	KSWMESEFR	HLA-A*31:01	0.007	8	150	158
22	KSWMESEFR	HLA-A*31:01	0.007	8	150	158
23	WMESEFRVY	HLA-A*30:02	0	8	152	160
23	WMESEFRVY	HLA-A*30:02	0	8	152	160
24	RVYSSANNC	HLA-B*57:01	0.01	8	158	166
24	RVYSSANNC	HLA-B*57:01	0.01	8	158	166
25	YSSANNCTF	HLA-B*15:01	0.003	8	160	168
25	YSSANNCTF	HLA-B*58:01	0.003	8	160	168
25	YSSANNCTF	HLA-B*15:01	0.003	8	160	168
25	YSSANNCTF	HLA-B*58:01	0.003	8	160	168
26	SANNCTFEY	HLA-B*35:01	0.002	8	162	170
26	SANNCTFEY	HLA-A*11:01	0.008	8	162	170
26	SANNCTFEY	HLA-A*30:02	0.003	8	162	170
26	SANNCTFEY	HLA-B*35:01	0.002	8	162	170
26	SANNCTFEY	HLA-A*11:01	0.008	8	162	170
26	SANNCTFEY	HLA-A*30:02	0.003	8	162	170
27	FEYVSQPFL	HLA-B*40:01	0.004	8	168	176
27	FEYVSQPFL	HLA-B*40:01	0.004	8	168	176
28	DLEGKQGNF	HLA-B*08:01	0.008	8	178	186
28	DLEGKQGNF	HLA-B*08:01	0.008	8	178	186
29	KQGNFKNLR	HLA-A*31:01	0.009	8	182	190
29	KQGNFKNLR	HLA-A*31:01	0.009	8	182	190
30	FVFKNIDGY	HLA-A*01:01	0.005	8	192	200
30	FVFKNIDGY	HLA-B*35:01	0.009	8	192	200
30	FVFKNIDGY	HLA-B*15:01	0.009	8	192	200
30	FVFKNIDGY	HLA-A*26:01	0.001	8	192	200
30	FVFKNIDGY	HLA-A*01:01	0.005	8	192	200
30	FVFKNIDGY	HLA-B*35:01	0.009	8	192	200
30	FVFKNIDGY	HLA-B*15:01	0.009	8	192	200

30	FVFKNIDGY	HLA-A*26:01	0.001	8	192	200
31	VFKNIDGYF	HLA-A*23:01	0.007	8	193	201
31	VFKNIDGYF	HLA-A*23:01	0.007	8	193	201
32	KIYSKHTPI	HLA-A*02:01	0.006	8	202	210
32	KIYSKHTPI	HLA-A*02:03	0.001	8	202	210
32	KIYSKHTPI	HLA-A*02:06	0.001	8	202	210
32	KIYSKHTPI	HLA-A*32:01	0	8	202	210
32	KIYSKHTPI	HLA-A*02:01	0.006	8	202	210
32	KIYSKHTPI	HLA-A*02:03	0.001	8	202	210
32	KIYSKHTPI	HLA-A*02:06	0.001	8	202	210
32	KIYSKHTPI	HLA-A*32:01	0	8	202	210
33	TPINLVRDL	HLA-B*07:02	0.008	8	208	216
33	TPINLVRDL	HLA-B*53:01	0.004	8	208	216
33	TPINLVRDL	HLA-B*07:02	0.008	8	208	216
33	TPINLVRDL	HLA-B*53:01	0.004	8	208	216
34	LVRDLPQGF	HLA-B*15:01	0.006	8	212	220
34	LVRDLPQGF	HLA-A*30:02	0.007	8	212	220
34	LVRDLPQGF	HLA-B*57:01	0.005	8	212	220
34	LVRDLPQGF	HLA-B*15:01	0.006	8	212	220
34	LVRDLPQGF	HLA-A*30:02	0.007	8	212	220
34	LVRDLPQGF	HLA-B*57:01	0.005	8	212	220
35	DLPQGFSAL	HLA-B*08:01	0.005	8	215	223
35	DLPQGFSAL	HLA-A*26:01	0.005	8	215	223
35	DLPQGFSAL	HLA-B*08:01	0.005	8	215	223
35	DLPQGFSAL	HLA-A*26:01	0.005	8	215	223
36	INITRFQTL	HLA-B*08:01	0.003	8	233	241
36	INITRFQTL	HLA-B*08:01	0.003	8	233	241
37	ITRFQTLLA	HLA-A*30:01	0.001	8	235	243
37	ITRFQTLLA	HLA-A*30:01	0.001	8	235	243
38	TPGDSSSGW	HLA-B*35:01	0.007	8	250	258
38	TPGDSSSGW	HLA-B*35:01	0.007	8	250	258
39	SSSGWTAGA	HLA-A*68:02	0.01	8	254	262
39	SSSGWTAGA	HLA-A*68:02	0.01	8	254	262
40	WTAGAAAYY	HLA-A*01:01	0.002	8	258	266
40	WTAGAAAYY	HLA-A*26:01	0.003	8	258	266
40	WTAGAAAYY	HLA-A*01:01	0.002	8	258	266
40	WTAGAAAYY	HLA-A*26:01	0.003	8	258	266
41	YYVGYLQPR	HLA-A*33:01	0.003	8	265	273
41	YYVGYLQPR	HLA-A*33:01	0.003	8	265	273
42	VGYLQPRTF	HLA-B*51:01	0.008	8	267	275
42	VGYLQPRTF	HLA-B*51:01	0.008	8	267	275
43	YLQPRTFLL	HLA-A*02:01	0.002	8	269	277
43	YLQPRTFLL	HLA-A*02:03	0.008	8	269	277
43	YLQPRTFLL	HLA-A*02:06	0.01	8	269	277
43	YLQPRTFLL	HLA-A*68:02	0.008	8	269	277
43	YLQPRTFLL	HLA-A*02:01	0.002	8	269	277
43	YLQPRTFLL	HLA-A*02:03	0.008	8	269	277
43	YLQPRTFLL	HLA-A*02:06	0.01	8	269	277
43	YLQPRTFLL	HLA-A*68:02	0.008	8	269	277
44	QPRTFLLKY	HLA-B*35:01	0.003	8	271	279

44	QPRTFLLKY	HLA-B*53:01	0.002	8	271	279
44	QPRTFLLKY	HLA-B*35:01	0.003	8	271	279
44	QPRTFLLKY	HLA-B*53:01	0.002	8	271	279
45	ENGTITDAV	HLA-B*08:01	0.008	8	281	289
45	ENGTITDAV	HLA-B*08:01	0.008	8	281	289
46	GTITDAVDC	HLA-B*57:01	0.01	8	283	291
46	GTITDAVDC	HLA-B*57:01	0.01	8	283	291
47	ITDAVDCAL	HLA-B*58:01	0.006	8	285	293
47	ITDAVDCAL	HLA-A*32:01	0.001	8	285	293
47	ITDAVDCAL	HLA-B*58:01	0.006	8	285	293
47	ITDAVDCAL	HLA-A*32:01	0.001	8	285	293
48	AVDCALDPL	HLA-A*02:06	0.006	8	288	296
48	AVDCALDPL	HLA-A*02:06	0.006	8	288	296
49	ALDPLSETK	HLA-A*03:01	0.005	8	292	300
49	ALDPLSETK	HLA-A*11:01	0.005	8	292	300
49	ALDPLSETK	HLA-A*03:01	0.005	8	292	300
49	ALDPLSETK	HLA-A*11:01	0.005	8	292	300
50	ETKCTLKSF	HLA-A*26:01	0	8	298	306
50	ETKCTLKSF	HLA-A*26:01	0	8	298	306
51	KSFTVEKGI	HLA-B*58:01	0.005	8	304	312
51	KSFTVEKGI	HLA-A*32:01	0.01	8	304	312
51	KSFTVEKGI	HLA-B*57:01	0.002	8	304	312
51	KSFTVEKGI	HLA-B*58:01	0.005	8	304	312
51	KSFTVEKGI	HLA-A*32:01	0.01	8	304	312
51	KSFTVEKGI	HLA-B*57:01	0.002	8	304	312
52	GIYQTSNFR	HLA-A*03:01	0.003	8	311	319
52	GIYQTSNFR	HLA-A*11:01	0.004	8	311	319
52	GIYQTSNFR	HLA-A*31:01	0.009	8	311	319
52	GIYQTSNFR	HLA-A*03:01	0.003	8	311	319
52	GIYQTSNFR	HLA-A*11:01	0.004	8	311	319
52	GIYQTSNFR	HLA-A*31:01	0.009	8	311	319
53	RVQPTESIV	HLA-A*30:01	0.007	8	319	327
53	RVQPTESIV	HLA-A*30:01	0.007	8	319	327
54	QPTESIVRF	HLA-B*35:01	0.003	8	321	329
54	QPTESIVRF	HLA-B*53:01	0.002	8	321	329
54	QPTESIVRF	HLA-B*35:01	0.003	8	321	329
54	QPTESIVRF	HLA-B*53:01	0.002	8	321	329
55	NLCPFGEVF	HLA-B*15:01	0.009	8	334	342
55	NLCPFGEVF	HLA-A*32:01	0.005	8	334	342
55	NLCPFGEVF	HLA-B*15:01	0.009	8	334	342
55	NLCPFGEVF	HLA-A*32:01	0.005	8	334	342
56	GEVFNATRF	HLA-B*40:01	0.002	8	339	347
56	GEVFNATRF	HLA-B*40:01	0.002	8	339	347
57	FNATRFASV	HLA-B*08:01	0.001	8	342	350
57	FNATRFASV	HLA-B*08:01	0.001	8	342	350
58	ATRFASVYA	HLA-A*30:01	0.004	8	344	352
58	ATRFASVYA	HLA-A*30:01	0.004	8	344	352
59	FASVYAWNR	HLA-A*33:01	0.001	8	347	355
59	FASVYAWNR	HLA-A*33:01	0.001	8	347	355
60	VYAWNRKRI	HLA-A*24:02	0.003	8	350	358

60	VYAWNRKRI	HLA-A*23:01	0.003	8	350	358
60	VYAWNRKRI	HLA-A*24:02	0.003	8	350	358
60	VYAWNRKRI	HLA-A*23:01	0.003	8	350	358
61	NRKRISNCV	HLA-B*08:01	0.006	8	354	362
61	NRKRISNCV	HLA-B*08:01	0.006	8	354	362
62	RISNCVADY	HLA-B*15:01	0.007	8	357	365
62	RISNCVADY	HLA-B*58:01	0.005	8	357	365
62	RISNCVADY	HLA-B*15:01	0.007	8	357	365
62	RISNCVADY	HLA-B*58:01	0.005	8	357	365
63	CVADYSVLY	HLA-A*01:01	0.003	8	361	369
63	CVADYSVLY	HLA-A*03:01	0.005	8	361	369
63	CVADYSVLY	HLA-A*26:01	0.005	8	361	369
63	CVADYSVLY	HLA-A*30:02	0.001	8	361	369
63	CVADYSVLY	HLA-A*01:01	0.003	8	361	369
63	CVADYSVLY	HLA-A*03:01	0.005	8	361	369
63	CVADYSVLY	HLA-A*26:01	0.005	8	361	369
63	CVADYSVLY	HLA-A*30:02	0.001	8	361	369
64	SVLYNSASF	HLA-A*32:01	0.004	8	366	374
64	SVLYNSASF	HLA-A*23:01	0.009	8	366	374
64	SVLYNSASF	HLA-A*32:01	0.004	8	366	374
64	SVLYNSASF	HLA-A*23:01	0.009	8	366	374
65	FSTFKCYGV	HLA-A*68:02	0.006	8	374	382
65	FSTFKCYGV	HLA-A*68:02	0.006	8	374	382
66	CYGVSPTKL	HLA-A*24:02	0.005	8	379	387
66	CYGVSPTKL	HLA-A*23:01	0	8	379	387
66	CYGVSPTKL	HLA-A*24:02	0.005	8	379	387
66	CYGVSPTKL	HLA-A*23:01	0	8	379	387
67	DEVRQIAPG	HLA-B*44:03	0.009	8	405	413
67	DEVRQIAPG	HLA-B*44:03	0.009	8	405	413
68	QIAPGQTGK	HLA-A*03:01	0.003	8	409	417
68	QIAPGQTGK	HLA-A*68:01	0.006	8	409	417
68	QIAPGQTGK	HLA-A*03:01	0.003	8	409	417
68	QIAPGQTGK	HLA-A*68:01	0.006	8	409	417
69	APGQTGKIA	HLA-B*07:02	0.007	8	411	419
69	APGQTGKIA	HLA-B*07:02	0.007	8	411	419
70	KIADYNYKL	HLA-A*02:01	0.001	8	417	425
70	KIADYNYKL	HLA-A*02:03	0.005	8	417	425
70	KIADYNYKL	HLA-A*02:06	0.002	8	417	425
70	KIADYNYKL	HLA-A*68:02	0.003	8	417	425
70	KIADYNYKL	HLA-A*32:01	0.006	8	417	425
70	KIADYNYKL	HLA-A*02:01	0.001	8	417	425
70	KIADYNYKL	HLA-A*02:03	0.005	8	417	425
70	KIADYNYKL	HLA-A*02:06	0.002	8	417	425
70	KIADYNYKL	HLA-A*68:02	0.003	8	417	425
70	KIADYNYKL	HLA-A*32:01	0.006	8	417	425
71	LPDDFTGCV	HLA-B*07:02	0.002	8	425	433
71	LPDDFTGCV	HLA-B*07:02	0.002	8	425	433
72	KVGGNYNYL	HLA-A*02:06	0.008	8	444	452
72	KVGGNYNYL	HLA-A*02:06	0.008	8	444	452
73	VGGNYNYLY	HLA-B*57:01	0.003	8	445	453

73	VGGNYNYLY	HLA-B*57:01	0.003	8	445	453
74	RLFRKSNLK	HLA-A*03:01	0.002	8	454	462
74	RLFRKSNLK	HLA-A*11:01	0.008	8	454	462
74	RLFRKSNLK	HLA-A*03:01	0.002	8	454	462
74	RLFRKSNLK	HLA-A*11:01	0.008	8	454	462
75	KSNLKPFER	HLA-A*31:01	0.001	8	458	466
75	KSNLKPFER	HLA-A*31:01	0.001	8	458	466
76	KPFERDIST	HLA-B*35:01	0.002	8	462	470
76	KPFERDIST	HLA-B*07:02	0.004	8	462	470
76	KPFERDIST	HLA-B*35:01	0.002	8	462	470
76	KPFERDIST	HLA-B*07:02	0.004	8	462	470
77	TPCNGVEGF	HLA-B*35:01	0.006	8	478	486
77	TPCNGVEGF	HLA-B*51:01	0.008	8	478	486
77	TPCNGVEGF	HLA-B*35:01	0.006	8	478	486
77	TPCNGVEGF	HLA-B*51:01	0.008	8	478	486
78	YGFQPTNGV	HLA-A*68:02	0.003	8	495	503
78	YGFQPTNGV	HLA-A*68:02	0.003	8	495	503
79	FQPTNGVGY	HLA-B*15:01	0.01	8	497	505
79	FQPTNGVGY	HLA-B*15:01	0.01	8	497	505
80	PYRVVVLSF	HLA-A*24:02	0.009	8	507	515
80	PYRVVVLSF	HLA-A*23:01	0.001	8	507	515
80	PYRVVVLSF	HLA-A*24:02	0.009	8	507	515
80	PYRVVVLSF	HLA-A*23:01	0.001	8	507	515
81	GPKKSTNLV	HLA-B*07:02	0.009	8	526	534
81	GPKKSTNLV	HLA-B*07:02	0.009	8	526	534
82	LVKNKCVNF	HLA-B*08:01	0.01	8	533	541
82	LVKNKCVNF	HLA-B*15:01	0.01	8	533	541
82	LVKNKCVNF	HLA-A*26:01	0.008	8	533	541
82	LVKNKCVNF	HLA-B*08:01	0.01	8	533	541
82	LVKNKCVNF	HLA-B*15:01	0.01	8	533	541
82	LVKNKCVNF	HLA-A*26:01	0.008	8	533	541
83	CVNFNFNGL	HLA-A*68:02	0.003	8	538	546
83	CVNFNFNGL	HLA-A*30:02	0.005	8	538	546
83	CVNFNFNGL	HLA-A*68:02	0.003	8	538	546
83	CVNFNFNGL	HLA-A*30:02	0.005	8	538	546
84	GVLTESNKK	HLA-A*03:01	0.009	8	550	558
84	GVLTESNKK	HLA-A*11:01	0.009	8	550	558
84	GVLTESNKK	HLA-A*03:01	0.009	8	550	558
84	GVLTESNKK	HLA-A*11:01	0.009	8	550	558
85	ESNKKFLPF	HLA-B*08:01	0.004	8	554	562
85	ESNKKFLPF	HLA-B*15:01	0.006	8	554	562
85	ESNKKFLPF	HLA-A*26:01	0.005	8	554	562
85	ESNKKFLPF	HLA-B*08:01	0.004	8	554	562
85	ESNKKFLPF	HLA-B*15:01	0.006	8	554	562
85	ESNKKFLPF	HLA-A*26:01	0.005	8	554	562
86	DIADTTDAV	HLA-A*68:02	0.003	8	568	576
86	DIADTTDAV	HLA-A*68:02	0.003	8	568	576
87	ITPCSFGGV	HLA-A*68:02	0.009	8	587	595
87	ITPCSFGGV	HLA-A*26:01	0.01	8	587	595
87	ITPCSFGGV	HLA-A*68:02	0.009	8	587	595

87	ITPCSFGGV	HLA-A*26:01	0.01	8	587	595
88	TSNQVAVLY	HLA-A*01:01	0.006	8	604	612
88	TSNQVAVLY	HLA-A*01:01	0.006	8	604	612
89	YQDVNCTEV	HLA-A*02:01	0.004	8	612	620
89	YQDVNCTEV	HLA-A*02:06	0.001	8	612	620
89	YQDVNCTEV	HLA-A*02:01	0.004	8	612	620
89	YQDVNCTEV	HLA-A*02:06	0.001	8	612	620
90	DVNCTEVPV	HLA-A*68:02	0.009	8	614	622
90	DVNCTEVPV	HLA-A*68:02	0.009	8	614	622
91	CTEVPVAIH	HLA-A*01:01	0.01	8	617	625
91	CTEVPVAIH	HLA-A*01:01	0.01	8	617	625
92	HADQLTPTW	HLA-B*58:01	0.003	8	625	633
92	HADQLTPTW	HLA-B*53:01	0.007	8	625	633
92	HADQLTPTW	HLA-B*58:01	0.003	8	625	633
92	HADQLTPTW	HLA-B*53:01	0.007	8	625	633
93	VYSTGSNVF	HLA-A*24:02	0.003	8	635	643
93	VYSTGSNVF	HLA-A*24:02	0.003	8	635	643
94	QTRAGCLIG	HLA-A*30:01	0.006	8	644	652
94	QTRAGCLIG	HLA-A*30:01	0.006	8	644	652
95	GAEHVNNSY	HLA-A*01:01	0.003	8	652	660
95	GAEHVNNSY	HLA-A*01:01	0.003	8	652	660
96	IPIGAGICA	HLA-B*07:02	0.004	8	664	672
96	IPIGAGICA	HLA-B*07:02	0.004	8	664	672
97	IGAGICASY	HLA-A*01:01	0.007	8	666	674
97	IGAGICASY	HLA-B*58:01	0.006	8	666	674
97	IGAGICASY	HLA-A*26:01	0.006	8	666	674
97	IGAGICASY	HLA-A*30:02	0.001	8	666	674
97	IGAGICASY	HLA-A*01:01	0.007	8	666	674
97	IGAGICASY	HLA-B*58:01	0.006	8	666	674
97	IGAGICASY	HLA-A*26:01	0.006	8	666	674
97	IGAGICASY	HLA-A*30:02	0.001	8	666	674
98	SPRRARSVA	HLA-B*07:02	0.006	8	680	688
98	SPRRARSVA	HLA-B*07:02	0.006	8	680	688
99	VASQSIIAY	HLA-A*01:01	0.009	8	687	695
99	VASQSIIAY	HLA-B*35:01	0.007	8	687	695
99	VASQSIIAY	HLA-A*01:01	0.009	8	687	695
99	VASQSIIAY	HLA-B*35:01	0.007	8	687	695
100	MSLGAENSV	HLA-B*57:01	0.002	8	697	705
100	MSLGAENSV	HLA-B*57:01	0.002	8	697	705
101	LGAENSVAY	HLA-A*01:01	0.007	8	699	707
101	LGAENSVAY	HLA-B*35:01	0.001	8	699	707
101	LGAENSVAY	HLA-A*01:01	0.007	8	699	707
101	LGAENSVAY	HLA-B*35:01	0.001	8	699	707
102	AENSVAYSN	HLA-B*44:03	0.008	8	701	709
102	AENSVAYSN	HLA-B*44:03	0.008	8	701	709
103	SVAYSNNSI	HLA-B*07:02	0.009	8	704	712
103	SVAYSNNSI	HLA-A*32:01	0.009	8	704	712
103	SVAYSNNSI	HLA-B*07:02	0.009	8	704	712
103	SVAYSNNSI	HLA-A*32:01	0.009	8	704	712
104	IAIPTNFTI	HLA-B*58:01	0.001	8	712	720

104	IAIPTNFTI	HLA-B*51:01	0.006	8	712	720
104	IAIPTNFTI	HLA-B*53:01	0.01	8	712	720
104	IAIPTNFTI	HLA-B*57:01	0.006	8	712	720
104	IAIPTNFTI	HLA-B*58:01	0.001	8	712	720
104	IAIPTNFTI	HLA-B*51:01	0.006	8	712	720
104	IAIPTNFTI	HLA-B*53:01	0.01	8	712	720
104	IAIPTNFTI	HLA-B*57:01	0.006	8	712	720
105	IPTNFTISV	HLA-B*07:02	0.006	8	714	722
105	IPTNFTISV	HLA-B*07:02	0.006	8	714	722
106	FTISVTTEI	HLA-A*02:03	0.01	8	718	726
106	FTISVTTEI	HLA-A*02:06	0.006	8	718	726
106	FTISVTTEI	HLA-A*68:02	0.008	8	718	726
106	FTISVTTEI	HLA-A*02:03	0.01	8	718	726
106	FTISVTTEI	HLA-A*02:06	0.006	8	718	726
106	FTISVTTEI	HLA-A*68:02	0.008	8	718	726
107	TEILPVSMT	HLA-B*44:02	0.003	8	724	732
107	TEILPVSMT	HLA-B*44:03	0.007	8	724	732
107	TEILPVSMT	HLA-B*44:02	0.003	8	724	732
107	TEILPVSMT	HLA-B*44:03	0.007	8	724	732
108	EILPVSMTK	HLA-A*11:01	0.009	8	725	733
108	EILPVSMTK	HLA-A*68:01	0.009	8	725	733
108	EILPVSMTK	HLA-A*33:01	0.005	8	725	733
108	EILPVSMTK	HLA-A*11:01	0.009	8	725	733
108	EILPVSMTK	HLA-A*68:01	0.009	8	725	733
108	EILPVSMTK	HLA-A*33:01	0.005	8	725	733
109	KTSVDCTMY	HLA-A*01:01	0.006	8	733	741
109	KTSVDCTMY	HLA-A*30:02	0	8	733	741
109	KTSVDCTMY	HLA-A*01:01	0.006	8	733	741
109	KTSVDCTMY	HLA-A*30:02	0	8	733	741
110	ECSNLLLQY	HLA-A*26:01	0.003	8	748	756
110	ECSNLLLQY	HLA-B*57:01	0.01	8	748	756
110	ECSNLLLQY	HLA-A*26:01	0.003	8	748	756
110	ECSNLLLQY	HLA-B*57:01	0.01	8	748	756
111	QYGSFCTQL	HLA-A*24:02	0.003	8	755	763
111	QYGSFCTQL	HLA-A*23:01	0.002	8	755	763
111	QYGSFCTQL	HLA-A*24:02	0.003	8	755	763
111	QYGSFCTQL	HLA-A*23:01	0.002	8	755	763
112	GSFCTQLNR	HLA-A*03:01	0.01	8	757	765
112	GSFCTQLNR	HLA-A*11:01	0.002	8	757	765
112	GSFCTQLNR	HLA-A*03:01	0.01	8	757	765
112	GSFCTQLNR	HLA-A*11:01	0.002	8	757	765
113	QLNRALTGI	HLA-A*02:03	0.004	8	762	770
113	QLNRALTGI	HLA-A*02:06	0.006	8	762	770
113	QLNRALTGI	HLA-A*68:02	0.004	8	762	770
113	QLNRALTGI	HLA-A*02:03	0.004	8	762	770
113	QLNRALTGI	HLA-A*02:06	0.006	8	762	770
113	QLNRALTGI	HLA-A*68:02	0.004	8	762	770
114	EQDKNTQEV	HLA-A*01:01	0.006	8	773	781
114	EQDKNTQEV	HLA-A*02:01	0.01	8	773	781
114	EQDKNTQEV	HLA-A*02:06	0.003	8	773	781

114	EQDKNTQEV	HLA-A*01:01	0.006	8	773	781
114	EQDKNTQEV	HLA-A*02:01	0.01	8	773	781
114	EQDKNTQEV	HLA-A*02:06	0.003	8	773	781
115	QDKNTQEVF	HLA-B*08:01	0.01	8	774	782
115	QDKNTQEVF	HLA-B*08:01	0.01	8	774	782
116	NTQEVFAQV	HLA-A*02:03	0.009	8	777	785
116	NTQEVFAQV	HLA-A*68:02	0	8	777	785
116	NTQEVFAQV	HLA-A*02:03	0.009	8	777	785
116	NTQEVFAQV	HLA-A*68:02	0	8	777	785
117	FAQVKQIYK	HLA-A*33:01	0.007	8	782	790
117	FAQVKQIYK	HLA-A*33:01	0.007	8	782	790
118	KQIYKTPPI	HLA-A*02:01	0.006	8	786	794
118	KQIYKTPPI	HLA-A*02:03	0.008	8	786	794
118	KQIYKTPPI	HLA-A*02:06	0.001	8	786	794
118	KQIYKTPPI	HLA-A*32:01	0	8	786	794
118	KQIYKTPPI	HLA-A*02:01	0.006	8	786	794
118	KQIYKTPPI	HLA-A*02:03	0.008	8	786	794
118	KQIYKTPPI	HLA-A*02:06	0.001	8	786	794
118	KQIYKTPPI	HLA-A*32:01	0	8	786	794
119	FGGFNFSQI	HLA-B*51:01	0.009	8	797	805
119	FGGFNFSQI	HLA-B*51:01	0.009	8	797	805
120	RSFIEDLLF	HLA-B*58:01	0	8	815	823
120	RSFIEDLLF	HLA-B*57:01	0	8	815	823
120	RSFIEDLLF	HLA-B*58:01	0	8	815	823
120	RSFIEDLLF	HLA-B*57:01	0	8	815	823
121	KVTLADAGF	HLA-B*58:01	0.004	8	825	833
121	KVTLADAGF	HLA-B*57:01	0.003	8	825	833
121	KVTLADAGF	HLA-B*58:01	0.004	8	825	833
121	KVTLADAGF	HLA-B*57:01	0.003	8	825	833
122	TLADAGFIK	HLA-A*03:01	0.003	8	827	835
122	TLADAGFIK	HLA-A*11:01	0.005	8	827	835
122	TLADAGFIK	HLA-A*31:01	0.01	8	827	835
122	TLADAGFIK	HLA-A*68:01	0.006	8	827	835
122	TLADAGFIK	HLA-A*03:01	0.003	8	827	835
122	TLADAGFIK	HLA-A*11:01	0.005	8	827	835
122	TLADAGFIK	HLA-A*31:01	0.01	8	827	835
122	TLADAGFIK	HLA-A*68:01	0.006	8	827	835
123	ADAGFIKQY	HLA-B*44:02	0.004	8	829	837
123	ADAGFIKQY	HLA-B*44:03	0.004	8	829	837
123	ADAGFIKQY	HLA-B*44:02	0.004	8	829	837
123	ADAGFIKQY	HLA-B*44:03	0.004	8	829	837
124	FIKQYGDCL	HLA-B*07:02	0.004	8	833	841
124	FIKQYGDCL	HLA-B*08:01	0.003	8	833	841
124	FIKQYGDCL	HLA-B*07:02	0.004	8	833	841
124	FIKQYGDCL	HLA-B*08:01	0.003	8	833	841
125	QYGDCLGDI	HLA-A*24:02	0.002	8	836	844
125	QYGDCLGDI	HLA-A*23:01	0	8	836	844
125	QYGDCLGDI	HLA-A*24:02	0.002	8	836	844
125	QYGDCLGDI	HLA-A*23:01	0	8	836	844
126	LTDEMIAQY	HLA-A*01:01	0.001	8	865	873

126	LTDEMIAQY	HLA-A*03:01	0.008	8	865	873
126	LTDEMIAQY	HLA-B*58:01	0.007	8	865	873
126	LTDEMIAQY	HLA-A*30:02	0.009	8	865	873
126	LTDEMIAQY	HLA-A*01:01	0.001	8	865	873
126	LTDEMIAQY	HLA-A*03:01	0.008	8	865	873
126	LTDEMIAQY	HLA-B*58:01	0.007	8	865	873
126	LTDEMIAQY	HLA-A*30:02	0.009	8	865	873
127	MIAQYTSAL	HLA-B*07:02	0.006	8	869	877
127	MIAQYTSAL	HLA-B*08:01	0.002	8	869	877
127	MIAQYTSAL	HLA-B*07:02	0.006	8	869	877
127	MIAQYTSAL	HLA-B*08:01	0.002	8	869	877
128	GTITSGWTF	HLA-B*15:01	0.005	8	880	888
128	GTITSGWTF	HLA-B*57:01	0.007	8	880	888
128	GTITSGWTF	HLA-B*15:01	0.005	8	880	888
128	GTITSGWTF	HLA-B*57:01	0.007	8	880	888
129	ITSGWTFGA	HLA-A*01:01	0.008	8	882	890
129	ITSGWTFGA	HLA-A*68:02	0.008	8	882	890
129	ITSGWTFGA	HLA-A*01:01	0.008	8	882	890
129	ITSGWTFGA	HLA-A*68:02	0.008	8	882	890
130	WTFGAGAAL	HLA-A*26:01	0.002	8	886	894
130	WTFGAGAAL	HLA-A*26:01	0.002	8	886	894
131	FGAGAALQI	HLA-B*51:01	0.008	8	888	896
131	FGAGAALQI	HLA-B*51:01	0.008	8	888	896
132	FAMQMAYRF	HLA-B*53:01	0.006	8	898	906
132	FAMQMAYRF	HLA-B*53:01	0.006	8	898	906
133	QMAYRFNGI	HLA-B*08:01	0.005	8	901	909
133	QMAYRFNGI	HLA-A*32:01	0.007	8	901	909
133	QMAYRFNGI	HLA-B*08:01	0.005	8	901	909
133	QMAYRFNGI	HLA-A*32:01	0.007	8	901	909
134	IGVTQNVLY	HLA-B*57:01	0.008	8	909	917
134	IGVTQNVLY	HLA-B*57:01	0.008	8	909	917
135	VLYENQKLI	HLA-A*02:03	0.006	8	915	923
135	VLYENQKLI	HLA-A*02:03	0.006	8	915	923
136	IANQFNSAI	HLA-B*53:01	0.009	8	923	931
136	IANQFNSAI	HLA-B*53:01	0.009	8	923	931
137	NQFNSAIGK	HLA-A*11:01	0.008	8	925	933
137	NQFNSAIGK	HLA-A*11:01	0.008	8	925	933
138	SSTASALGK	HLA-A*03:01	0.008	8	939	947
138	SSTASALGK	HLA-A*11:01	0.009	8	939	947
138	SSTASALGK	HLA-A*03:01	0.008	8	939	947
138	SSTASALGK	HLA-A*11:01	0.009	8	939	947
139	STASALGKL	HLA-A*68:02	0.008	8	940	948
139	STASALGKL	HLA-A*26:01	0.004	8	940	948
139	STASALGKL	HLA-A*68:02	0.008	8	940	948
139	STASALGKL	HLA-A*26:01	0.004	8	940	948
140	VVNQNAQAL	HLA-B*35:01	0.008	8	951	959
140	VVNQNAQAL	HLA-B*07:02	0.003	8	951	959
140	VVNQNAQAL	HLA-B*35:01	0.008	8	951	959
140	VVNQNAQAL	HLA-B*07:02	0.003	8	951	959
141	NAQALNTLV	HLA-A*68:02	0.01	8	955	963

141	NAQALNTLV	HLA-A*68:02	0.01	8	955	963
142	AQALNTLVK	HLA-A*03:01	0.01	8	956	964
142	AQALNTLVK	HLA-A*11:01	0.009	8	956	964
142	AQALNTLVK	HLA-A*03:01	0.01	8	956	964
142	AQALNTLVK	HLA-A*11:01	0.009	8	956	964
143	LVKQLSSNF	HLA-A*26:01	0.003	8	962	970
143	LVKQLSSNF	HLA-A*26:01	0.003	8	962	970
144	KQLSSNFGA	HLA-A*02:01	0.006	8	964	972
144	KQLSSNFGA	HLA-A*02:06	0.002	8	964	972
144	KQLSSNFGA	HLA-A*30:01	0.009	8	964	972
144	KQLSSNFGA	HLA-A*02:01	0.006	8	964	972
144	KQLSSNFGA	HLA-A*02:06	0.002	8	964	972
144	KQLSSNFGA	HLA-A*30:01	0.009	8	964	972
145	NFGAISSVL	HLA-A*23:01	0.004	8	969	977
145	NFGAISSVL	HLA-A*23:01	0.004	8	969	977
146	SVLNDILSR	HLA-A*03:01	0.01	8	975	983
146	SVLNDILSR	HLA-A*11:01	0.002	8	975	983
146	SVLNDILSR	HLA-A*31:01	0.005	8	975	983
146	SVLNDILSR	HLA-A*33:01	0.003	8	975	983
146	SVLNDILSR	HLA-A*03:01	0.01	8	975	983
146	SVLNDILSR	HLA-A*11:01	0.002	8	975	983
146	SVLNDILSR	HLA-A*31:01	0.005	8	975	983
146	SVLNDILSR	HLA-A*33:01	0.003	8	975	983
147	VLNDILSRL	HLA-A*02:03	0.001	8	976	984
147	VLNDILSRL	HLA-A*02:03	0.001	8	976	984
148	RLDKVEAEV	HLA-A*02:01	0	8	983	991
148	RLDKVEAEV	HLA-A*02:06	0	8	983	991
148	RLDKVEAEV	HLA-A*02:01	0	8	983	991
148	RLDKVEAEV	HLA-A*02:06	0	8	983	991
149	AEVQIDRLI	HLA-B*40:01	0.004	8	989	997
149	AEVQIDRLI	HLA-B*44:02	0	8	989	997
149	AEVQIDRLI	HLA-B*44:03	0.003	8	989	997
149	AEVQIDRLI	HLA-B*40:01	0.004	8	989	997
149	AEVQIDRLI	HLA-B*44:02	0	8	989	997
149	AEVQIDRLI	HLA-B*44:03	0.003	8	989	997
150	RLQSLQTYV	HLA-A*02:01	0.002	8	1000	1008
150	RLQSLQTYV	HLA-A*02:03	0.001	8	1000	1008
150	RLQSLQTYV	HLA-A*02:06	0.003	8	1000	1008
150	RLQSLQTYV	HLA-A*02:01	0.002	8	1000	1008
150	RLQSLQTYV	HLA-A*02:03	0.001	8	1000	1008
150	RLQSLQTYV	HLA-A*02:06	0.003	8	1000	1008
151	AEIRASANL	HLA-B*40:01	0.002	8	1016	1024
151	AEIRASANL	HLA-B*44:02	0.01	8	1016	1024
151	AEIRASANL	HLA-B*44:03	0.004	8	1016	1024
151	AEIRASANL	HLA-B*40:01	0.002	8	1016	1024
151	AEIRASANL	HLA-B*44:02	0.01	8	1016	1024
151	AEIRASANL	HLA-B*44:03	0.004	8	1016	1024
152	ASANLAATK	HLA-A*11:01	0.008	8	1020	1028
152	ASANLAATK	HLA-A*11:01	0.008	8	1020	1028
153	ATKMSECVL	HLA-A*30:01	0.007	8	1026	1034

153	ATKMSECVL	HLA-A*30:01	0.007	8	1026	1034
154	CVLGQSKRV	HLA-A*68:02	0.01	8	1032	1040
154	CVLGQSKRV	HLA-A*68:02	0.01	8	1032	1040
155	RVDFCGKGY	HLA-A*01:01	0.001	8	1039	1047
155	RVDFCGKGY	HLA-B*58:01	0.009	8	1039	1047
155	RVDFCGKGY	HLA-A*01:01	0.001	8	1039	1047
155	RVDFCGKGY	HLA-B*58:01	0.009	8	1039	1047
156	HLMSFPQSA	HLA-A*02:01	0.006	8	1048	1056
156	HLMSFPQSA	HLA-A*02:03	0.01	8	1048	1056
156	HLMSFPQSA	HLA-A*02:01	0.006	8	1048	1056
156	HLMSFPQSA	HLA-A*02:03	0.01	8	1048	1056
157	MSFPQSAPH	HLA-A*03:01	0.004	8	1050	1058
157	MSFPQSAPH	HLA-A*03:01	0.004	8	1050	1058
158	FPQSAPHGV	HLA-B*51:01	0.009	8	1052	1060
158	FPQSAPHGV	HLA-B*51:01	0.009	8	1052	1060
159	QSAPHGVVF	HLA-B*15:01	0.005	8	1054	1062
159	QSAPHGVVF	HLA-B*15:01	0.005	8	1054	1062
160	VTYVPAQEK	HLA-A*03:01	0	8	1065	1073
160	VTYVPAQEK	HLA-A*11:01	0.003	8	1065	1073
160	VTYVPAQEK	HLA-A*31:01	0.005	8	1065	1073
160	VTYVPAQEK	HLA-A*68:01	0.006	8	1065	1073
160	VTYVPAQEK	HLA-A*33:01	0.007	8	1065	1073
160	VTYVPAQEK	HLA-A*03:01	0	8	1065	1073
160	VTYVPAQEK	HLA-A*11:01	0.003	8	1065	1073
160	VTYVPAQEK	HLA-A*31:01	0.005	8	1065	1073
160	VTYVPAQEK	HLA-A*68:01	0.006	8	1065	1073
160	VTYVPAQEK	HLA-A*33:01	0.007	8	1065	1073
161	KAHFPREGV	HLA-A*68:02	0.001	8	1086	1094
161	KAHFPREGV	HLA-A*68:02	0.001	8	1086	1094
162	REGVFVSNG	HLA-B*44:02	0.009	8	1091	1099
162	REGVFVSNG	HLA-B*44:03	0.004	8	1091	1099
162	REGVFVSNG	HLA-B*44:02	0.009	8	1091	1099
162	REGVFVSNG	HLA-B*44:03	0.004	8	1091	1099
163	VFVSNGTHW	HLA-A*23:01	0.009	8	1094	1102
163	VFVSNGTHW	HLA-A*23:01	0.009	8	1094	1102
164	FVSNGTHWF	HLA-A*01:01	0.008	8	1095	1103
164	FVSNGTHWF	HLA-B*35:01	0.004	8	1095	1103
164	FVSNGTHWF	HLA-B*53:01	0.007	8	1095	1103
164	FVSNGTHWF	HLA-A*26:01	0.007	8	1095	1103
164	FVSNGTHWF	HLA-A*01:01	0.008	8	1095	1103
164	FVSNGTHWF	HLA-B*35:01	0.004	8	1095	1103
164	FVSNGTHWF	HLA-B*53:01	0.007	8	1095	1103
164	FVSNGTHWF	HLA-A*26:01	0.007	8	1095	1103
165	GTHWFVTQR	HLA-A*11:01	0.008	8	1099	1107
165	GTHWFVTQR	HLA-A*31:01	0.004	8	1099	1107
165	GTHWFVTQR	HLA-A*68:01	0.006	8	1099	1107
165	GTHWFVTQR	HLA-A*11:01	0.008	8	1099	1107
165	GTHWFVTQR	HLA-A*31:01	0.004	8	1099	1107
165	GTHWFVTQR	HLA-A*68:01	0.006	8	1099	1107
166	QIITTDNTF	HLA-B*15:01	0.004	8	1113	1121

166	QIITTDNTF	HLA-B*15:01	0.004	8	1113	1121
167	IGIVNNTVY	HLA-A*30:02	0.002	8	1130	1138
167	IGIVNNTVY	HLA-A*30:02	0.002	8	1130	1138
168	VYDPLQPEL	HLA-A*24:02	0.001	8	1137	1145
168	VYDPLQPEL	HLA-A*24:02	0.001	8	1137	1145
169	PLQPELDSF	HLA-A*23:01	0.002	8	1140	1148
169	PLQPELDSF	HLA-A*23:01	0.002	8	1140	1148
170	SFKEELDKY	HLA-A*26:01	0.007	8	1147	1155
170	SFKEELDKY	HLA-A*30:02	0.009	8	1147	1155
170	SFKEELDKY	HLA-A*26:01	0.007	8	1147	1155
170	SFKEELDKY	HLA-A*30:02	0.009	8	1147	1155
171	DISGINASV	HLA-A*68:02	0.001	8	1168	1176
171	DISGINASV	HLA-A*26:01	0.007	8	1168	1176
171	DISGINASV	HLA-A*68:02	0.001	8	1168	1176
171	DISGINASV	HLA-A*26:01	0.007	8	1168	1176
172	NASVVNIQK	HLA-A*68:01	0.008	8	1173	1181
172	NASVVNIQK	HLA-A*33:01	0.001	8	1173	1181
172	NASVVNIQK	HLA-A*68:01	0.008	8	1173	1181
172	NASVVNIQK	HLA-A*33:01	0.001	8	1173	1181
173	KEIDRLNEV	HLA-A*02:06	0.004	8	1181	1189
173	KEIDRLNEV	HLA-B*44:03	0.006	8	1181	1189
173	KEIDRLNEV	HLA-A*02:06	0.004	8	1181	1189
173	KEIDRLNEV	HLA-B*44:03	0.006	8	1181	1189
174	RLNEVAKNL	HLA-A*02:01	0.009	8	1185	1193
174	RLNEVAKNL	HLA-A*32:01	0.004	8	1185	1193
174	RLNEVAKNL	HLA-A*02:01	0.009	8	1185	1193
174	RLNEVAKNL	HLA-A*32:01	0.004	8	1185	1193
175	EVAKNLNES	HLA-A*68:02	0.007	8	1188	1196
175	EVAKNLNES	HLA-A*68:02	0.007	8	1188	1196
176	NLNESLIDL	HLA-A*02:03	0.007	8	1192	1200
176	NLNESLIDL	HLA-A*02:03	0.007	8	1192	1200
177	QELGKYEQY	HLA-B*44:02	0.01	8	1201	1209
177	QELGKYEQY	HLA-B*44:02	0.01	8	1201	1209
178	QYIKWPWYI	HLA-A*24:02	0.003	8	1208	1216
178	QYIKWPWYI	HLA-A*23:01	0.002	8	1208	1216
178	QYIKWPWYI	HLA-A*24:02	0.003	8	1208	1216
178	QYIKWPWYI	HLA-A*23:01	0.002	8	1208	1216
179	WPWYIWLGF	HLA-B*35:01	0.005	8	1212	1220
179	WPWYIWLGF	HLA-B*35:01	0.005	8	1212	1220
180	FIAGLIAIV	HLA-A*02:03	0.007	8	1220	1228
180	FIAGLIAIV	HLA-A*02:06	0.01	8	1220	1228
180	FIAGLIAIV	HLA-A*68:02	0.007	8	1220	1228
180	FIAGLIAIV	HLA-A*02:03	0.007	8	1220	1228
180	FIAGLIAIV	HLA-A*02:06	0.01	8	1220	1228
180	FIAGLIAIV	HLA-A*68:02	0.007	8	1220	1228
181	CMTSCCSCL	HLA-A*23:01	0.007	8	1236	1244
181	CMTSCCSCL	HLA-A*30:02	0.009	8	1236	1244
181	CMTSCCSCL	HLA-A*23:01	0.007	8	1236	1244
181	CMTSCCSCL	HLA-A*30:02	0.009	8	1236	1244
182	MTSCCSCLK	HLA-A*03:01	0.006	8	1237	1245

182	MTSCCSCLK	HLA-A*11:01	0.002	8	1237	1245
182	MTSCCSCLK	HLA-A*30:01	0.004	8	1237	1245
182	MTSCCSCLK	HLA-A*68:01	0	8	1237	1245
182	MTSCCSCLK	HLA-A*03:01	0.006	8	1237	1245
182	MTSCCSCLK	HLA-A*11:01	0.002	8	1237	1245
182	MTSCCSCLK	HLA-A*30:01	0.004	8	1237	1245
182	MTSCCSCLK	HLA-A*68:01	0	8	1237	1245
183	EPVLKGVKL	HLA-B*35:01	0.002	8	1262	1270
183	EPVLKGVKL	HLA-B*53:01	0.001	8	1262	1270
183	EPVLKGVKL	HLA-B*35:01	0.002	8	1262	1270
183	EPVLKGVKL	HLA-B*53:01	0.001	8	1262	1270
184	VLKGVKLHY	HLA-B*15:01	0.002	8	1264	1272
184	VLKGVKLHY	HLA-B*15:01	0.002	8	1264	1272

Table S4. Vaxitop predicted MHC-II epitopes for SARS-CoV-2 S protein.

#	Epitope	MHC Allele	P-value	Length	Start	End
1	MFVFLVLLPLVSSQC	HLA-DRB1*08:02	0.004	14	1	15
1	MFVFLVLLPLVSSQC	HLA-DRB1*11:01	0.003	14	1	15
1	MFVFLVLLPLVSSQC	HLA-DRB1*08:02	0.004	14	1	15
1	MFVFLVLLPLVSSQC	HLA-DRB1*11:01	0.003	14	1	15
2	SSQCVNLTTRTQLPP	HLA-DRB1*12:01	0.001	14	12	26
2	SSQCVNLTTRTQLPP	HLA-DRB1*12:01	0.001	14	12	26
3	SQCVNLTTRTQLPPA	HLA-DQA1*01:02/DQB1*06:02	0.003	14	13	27
3	SQCVNLTTRTQLPPA	HLA-DQA1*01:02/DQB1*06:02	0.003	14	13	27
4	AYTNSFTRGVYYPDK	HLA-DQA1*05:01/DQB1*03:01	0.009	14	27	41
4	AYTNSFTRGVYYPDK	HLA-DQA1*05:01/DQB1*03:01	0.009	14	27	41
5	TNSFTRGVYYPDKVF	HLA-DRB3*01:01	0.005	14	29	43
5	TNSFTRGVYYPDKVF	HLA-DRB3*01:01	0.005	14	29	43
6	PDKVFRSSVLHSTQD	HLA-DRB1*01:01	0.004	14	39	53
6	PDKVFRSSVLHSTQD	HLA-DRB1*04:01	0.004	14	39	53
6	PDKVFRSSVLHSTQD	HLA-DRB5*01:01	0.005	14	39	53
6	PDKVFRSSVLHSTQD	HLA-DRB1*04:05	0.001	14	39	53
6	PDKVFRSSVLHSTQD	HLA-DRB1*07:01	0.009	14	39	53
6	PDKVFRSSVLHSTQD	HLA-DRB1*09:01	0.001	14	39	53
6	PDKVFRSSVLHSTQD	HLA-DRB1*15:01	0.003	14	39	53
6	PDKVFRSSVLHSTQD	HLA-DPA1*01:03/DPB1*04:01	0.006	14	39	53
6	PDKVFRSSVLHSTQD	HLA-DRB1*01:01	0.004	14	39	53
6	PDKVFRSSVLHSTQD	HLA-DRB1*04:01	0.004	14	39	53
6	PDKVFRSSVLHSTQD	HLA-DRB5*01:01	0.005	14	39	53
6	PDKVFRSSVLHSTQD	HLA-DRB1*04:05	0.001	14	39	53
6	PDKVFRSSVLHSTQD	HLA-DRB1*07:01	0.009	14	39	53
6	PDKVFRSSVLHSTQD	HLA-DRB1*09:01	0.001	14	39	53
6	PDKVFRSSVLHSTQD	HLA-DRB1*15:01	0.003	14	39	53
6	PDKVFRSSVLHSTQD	HLA-DPA1*01:03/DPB1*04:01	0.006	14	39	53
7	FRSSVLHSTQDLFLP	HLA-DQA1*05:01/DQB1*02:01	0.006	14	43	57
7	FRSSVLHSTQDLFLP	HLA-DQA1*05:01/DQB1*02:01	0.006	14	43	57
8	SNVTWFHAIHVSGTN	HLA-DPA1*02:01/DPB1*05:01	0.01	14	60	74
8	SNVTWFHAIHVSGTN	HLA-DPA1*02:01/DPB1*05:01	0.01	14	60	74
9	NVTWFHAIHVSGTNG	HLA-DRB1*09:01	0.007	14	61	75
9	NVTWFHAIHVSGTNG	HLA-DRB1*09:01	0.007	14	61	75
10	TWFHAIHVSGTNGTK	HLA-DPA1*01:03/DPB1*02:01	0.003	14	63	77
10	TWFHAIHVSGTNGTK	HLA-DPA1*01:03/DPB1*02:01	0.003	14	63	77
11	AIHVSGTNGTKRFDN	HLA-DQA1*05:01/DQB1*03:01	0.004	14	67	81
11	AIHVSGTNGTKRFDN	HLA-DQA1*05:01/DQB1*03:01	0.004	14	67	81
12	NGTKRFDNPVLPFND	HLA-DRB1*08:02	0.004	14	74	88
12	NGTKRFDNPVLPFND	HLA-DRB1*08:02	0.004	14	74	88
13	NDGVYFASTEKSNII	HLA-DRB1*07:01	0.002	14	87	101
13	NDGVYFASTEKSNII	HLA-DPA1*01:03/DPB1*02:01	0.001	14	87	101
13	NDGVYFASTEKSNII	HLA-DQA1*05:01/DQB1*02:01	0.005	14	87	101
13	NDGVYFASTEKSNII	HLA-DPA1*01:03/DPB1*04:01	0.004	14	87	101
13	NDGVYFASTEKSNII	HLA-DPA1*02:01/DPB1*01:01	0.001	14	87	101
13	NDGVYFASTEKSNII	HLA-DPA1*03:01/DPB1*04:02	0.009	14	87	101
13	NDGVYFASTEKSNII	HLA-DRB1*07:01	0.002	14	87	101

13	NDGVYFASTEKSNII	HLA-DPA1*01:03/DPB1*02:01	0.001	14	87	101
13	NDGVYFASTEKSNII	HLA-DQA1*05:01/DQB1*02:01	0.005	14	87	101
13	NDGVYFASTEKSNII	HLA-DPA1*01:03/DPB1*04:01	0.004	14	87	101
13	NDGVYFASTEKSNII	HLA-DPA1*02:01/DPB1*01:01	0.001	14	87	101
13	NDGVYFASTEKSNII	HLA-DPA1*03:01/DPB1*04:02	0.009	14	87	101
14	DGVYFASTEKSNIIR	HLA-DRB5*01:01	0.007	14	88	102
14	DGVYFASTEKSNIIR	HLA-DRB3*02:02	0.004	14	88	102
14	DGVYFASTEKSNIIR	HLA-DRB5*01:01	0.007	14	88	102
14	DGVYFASTEKSNIIR	HLA-DRB3*02:02	0.004	14	88	102
15	IRGWIFGTTLDSKTQ	HLA-DRB1*04:01	0.005	14	101	115
15	IRGWIFGTTLDSKTQ	HLA-DRB1*04:05	0.005	14	101	115
15	IRGWIFGTTLDSKTQ	HLA-DRB1*04:01	0.005	14	101	115
15	IRGWIFGTTLDSKTQ	HLA-DRB1*04:05	0.005	14	101	115
16	GTTLDSKTQSLLIVN	HLA-DQA1*05:01/DQB1*03:01	0.002	14	107	121
16	GTTLDSKTQSLLIVN	HLA-DPA1*01:03/DPB1*04:01	0.003	14	107	121
16	GTTLDSKTQSLLIVN	HLA-DQA1*05:01/DQB1*03:01	0.002	14	107	121
16	GTTLDSKTQSLLIVN	HLA-DPA1*01:03/DPB1*04:01	0.003	14	107	121
17	QSLLIVNNATNVVIK	HLA-DRB1*13:02	0.006	14	115	129
17	QSLLIVNNATNVVIK	HLA-DRB1*13:02	0.006	14	115	129
18	CEFQFCNDPFLGVYY	HLA-DRB1*03:01	0.001	14	131	145
18	CEFQFCNDPFLGVYY	HLA-DRB1*09:01	0.008	14	131	145
18	CEFQFCNDPFLGVYY	HLA-DPA1*03:01/DPB1*04:02	0.009	14	131	145
18	CEFQFCNDPFLGVYY	HLA-DRB1*03:01	0.001	14	131	145
18	CEFQFCNDPFLGVYY	HLA-DRB1*09:01	0.008	14	131	145
18	CEFQFCNDPFLGVYY	HLA-DPA1*03:01/DPB1*04:02	0.009	14	131	145
19	FLGVYYHKNNKSWME	HLA-DRB1*04:01	0.01	14	140	154
19	FLGVYYHKNNKSWME	HLA-DRB3*02:02	0.001	14	140	154
19	FLGVYYHKNNKSWME	HLA-DRB1*04:01	0.01	14	140	154
19	FLGVYYHKNNKSWME	HLA-DRB3*02:02	0.001	14	140	154
20	YHKNNKSWMESEFRV	HLA-DQA1*01:02/DQB1*06:02	0	14	145	159
20	YHKNNKSWMESEFRV	HLA-DQA1*01:02/DQB1*06:02	0	14	145	159
21	KNNKSWMESEFRVYS	HLA-DRB1*03:01	0.005	14	147	161
21	KNNKSWMESEFRVYS	HLA-DRB1*03:01	0.005	14	147	161
22	MESEFRVYSSANNCT	HLA-DRB1*13:02	0.005	14	153	167
22	MESEFRVYSSANNCT	HLA-DPA1*01:03/DPB1*04:01	0.003	14	153	167
22	MESEFRVYSSANNCT	HLA-DPA1*02:01/DPB1*01:01	0.004	14	153	167
22	MESEFRVYSSANNCT	HLA-DPA1*03:01/DPB1*04:02	0.001	14	153	167
22	MESEFRVYSSANNCT	HLA-DRB1*13:02	0.005	14	153	167
22	MESEFRVYSSANNCT	HLA-DPA1*01:03/DPB1*04:01	0.003	14	153	167
22	MESEFRVYSSANNCT	HLA-DPA1*02:01/DPB1*01:01	0.004	14	153	167
22	MESEFRVYSSANNCT	HLA-DPA1*03:01/DPB1*04:02	0.001	14	153	167
23	ESEFRVYSSANNCTF	HLA-DRB1*04:05	0.005	14	154	168
23	ESEFRVYSSANNCTF	HLA-DRB1*04:05	0.005	14	154	168
24	NCTFEYVSQPFLMDL	HLA-DQA1*05:01/DQB1*03:01	0.001	14	165	179
24	NCTFEYVSQPFLMDL	HLA-DRB1*07:01	0.001	14	165	179
24	NCTFEYVSQPFLMDL	HLA-DQA1*05:01/DQB1*03:01	0.001	14	165	179
24	NCTFEYVSQPFLMDL	HLA-DRB1*07:01	0.001	14	165	179
25	FEYVSQPFLMDLEGK	HLA-DPA1*01:03/DPB1*04:01	0.009	14	168	182
25	FEYVSQPFLMDLEGK	HLA-DPA1*03:01/DPB1*04:02	0.006	14	168	182
25	FEYVSQPFLMDLEGK	HLA-DPA1*01:03/DPB1*04:01	0.009	14	168	182

25	FEYVSQPFLMDLEGK	HLA-DPA1*03:01/DPB1*04:02	0.006	14	168	182
26	NFKNLREFVFKNIDG	HLA-DQA1*01:02/DQB1*06:02	0.009	14	185	199
26	NFKNLREFVFKNIDG	HLA-DQA1*01:02/DQB1*06:02	0.009	14	185	199
27	REFVFKNIDGYFKIY	HLA-DRB1*13:02	0.005	14	190	204
27	REFVFKNIDGYFKIY	HLA-DRB1*13:02	0.005	14	190	204
28	IDGYFKIYSKHTPIN	HLA-DRB1*01:01	0.005	14	197	211
28	IDGYFKIYSKHTPIN	HLA-DRB1*09:01	0.003	14	197	211
28	IDGYFKIYSKHTPIN	HLA-DRB3*02:02	0.005	14	197	211
28	IDGYFKIYSKHTPIN	HLA-DRB1*01:01	0.005	14	197	211
28	IDGYFKIYSKHTPIN	HLA-DRB1*09:01	0.003	14	197	211
28	IDGYFKIYSKHTPIN	HLA-DRB3*02:02	0.005	14	197	211
29	DGYFKIYSKHTPINL	HLA-DRB1*07:01	0.001	14	198	212
29	DGYFKIYSKHTPINL	HLA-DRB1*11:01	0.007	14	198	212
29	DGYFKIYSKHTPINL	HLA-DRB1*07:01	0.001	14	198	212
29	DGYFKIYSKHTPINL	HLA-DRB1*11:01	0.007	14	198	212
30	YFKIYSKHTPINLVR	HLA-DRB5*01:01	0.006	14	200	214
30	YFKIYSKHTPINLVR	HLA-DRB5*01:01	0.006	14	200	214
31	FKIYSKHTPINLVRD	HLA-DRB1*04:01	0.002	14	201	215
31	FKIYSKHTPINLVRD	HLA-DQA1*05:01/DQB1*03:01	0.003	14	201	215
31	FKIYSKHTPINLVRD	HLA-DRB1*04:01	0.002	14	201	215
31	FKIYSKHTPINLVRD	HLA-DQA1*05:01/DQB1*03:01	0.003	14	201	215
32	YSKHTPINLVRDLPQ	HLA-DPA1*01:03/DPB1*02:01	0.007	14	204	218
32	YSKHTPINLVRDLPQ	HLA-DPA1*01:03/DPB1*02:01	0.007	14	204	218
33	PQGFSALEPLVDLPI	HLA-DRB4*01:01	0.007	14	217	231
33	PQGFSALEPLVDLPI	HLA-DRB4*01:01	0.007	14	217	231
34	QGFSALEPLVDLPIG	HLA-DPA1*02:01/DPB1*05:01	0.007	14	218	232
34	QGFSALEPLVDLPIG	HLA-DPA1*02:01/DPB1*05:01	0.007	14	218	232
35	PIGINITRFQTLLAL	HLA-DPA1*02:01/DPB1*01:01	0.003	14	230	244
35	PIGINITRFQTLLAL	HLA-DPA1*02:01/DPB1*01:01	0.003	14	230	244
36	ITRFQTLLALHRSYL	HLA-DRB5*01:01	0.001	14	235	249
36	ITRFQTLLALHRSYL	HLA-DRB1*04:05	0.009	14	235	249
36	ITRFQTLLALHRSYL	HLA-DRB5*01:01	0.001	14	235	249
36	ITRFQTLLALHRSYL	HLA-DRB1*04:05	0.009	14	235	249
37	TRFQTLLALHRSYLT	HLA-DRB3*01:01	0.01	14	236	250
37	TRFQTLLALHRSYLT	HLA-DRB3*01:01	0.01	14	236	250
38	FQTLLALHRSYLTPG	HLA-DRB1*03:01	0.008	14	238	252
38	FQTLLALHRSYLTPG	HLA-DQA1*05:01/DQB1*03:01	0.003	14	238	252
38	FQTLLALHRSYLTPG	HLA-DPA1*01:03/DPB1*04:01	0.01	14	238	252
38	FQTLLALHRSYLTPG	HLA-DRB1*03:01	0.008	14	238	252
38	FQTLLALHRSYLTPG	HLA-DQA1*05:01/DQB1*03:01	0.003	14	238	252
38	FQTLLALHRSYLTPG	HLA-DPA1*01:03/DPB1*04:01	0.01	14	238	252
39	GYLQPRTFLLKYNEN	HLA-DPA1*01:03/DPB1*04:01	0.003	14	268	282
39	GYLQPRTFLLKYNEN	HLA-DPA1*01:03/DPB1*04:01	0.003	14	268	282
40	PRTFLLKYNENGTIT	HLA-DRB1*04:05	0.008	14	272	286
40	PRTFLLKYNENGTIT	HLA-DRB1*13:02	0.008	14	272	286
40	PRTFLLKYNENGTIT	HLA-DRB1*04:05	0.008	14	272	286
40	PRTFLLKYNENGTIT	HLA-DRB1*13:02	0.008	14	272	286
41	RTFLLKYNENGTITD	HLA-DRB1*09:01	0.007	14	273	287
41	RTFLLKYNENGTITD	HLA-DRB1*09:01	0.007	14	273	287
42	LKYNENGTITDAVDC	HLA-DQA1*01:02/DQB1*06:02	0	14	277	291

42	LKYNENGTITDAVDC	HLA-DQA1*01:02/DQB1*06:02	0	14	277	291
43	YNENGTITDAVDCAL	HLA-DQA1*05:01/DQB1*02:01	0.006	14	279	293
43	YNENGTITDAVDCAL	HLA-DQA1*05:01/DQB1*02:01	0.006	14	279	293
44	AVDCALDPLSETKCT	HLA-DPA1*03:01/DPB1*04:02	0.007	14	288	302
44	AVDCALDPLSETKCT	HLA-DPA1*03:01/DPB1*04:02	0.007	14	288	302
45	DCALDPLSETKCTLK	HLA-DPA1*02:01/DPB1*01:01	0.008	14	290	304
45	DCALDPLSETKCTLK	HLA-DPA1*02:01/DPB1*01:01	0.008	14	290	304
46	KCTLKSFTVEKGIYQ	HLA-DRB1*04:05	0.004	14	300	314
46	KCTLKSFTVEKGIYQ	HLA-DRB1*07:01	0.003	14	300	314
46	KCTLKSFTVEKGIYQ	HLA-DRB1*04:05	0.004	14	300	314
46	KCTLKSFTVEKGIYQ	HLA-DRB1*07:01	0.003	14	300	314
47	EKGIYQTSNFRVQPT	HLA-DRB1*03:01	0.01	14	309	323
47	EKGIYQTSNFRVQPT	HLA-DRB4*01:01	0.002	14	309	323
47	EKGIYQTSNFRVQPT	HLA-DRB1*03:01	0.01	14	309	323
47	EKGIYQTSNFRVQPT	HLA-DRB4*01:01	0.002	14	309	323
48	YQTSNFRVQPTESIV	HLA-DQA1*05:01/DQB1*03:01	0.007	14	313	327
48	YQTSNFRVQPTESIV	HLA-DQA1*05:01/DQB1*03:01	0.007	14	313	327
49	ESIVRFPNITNLCPF	HLA-DRB3*02:02	0.009	14	324	338
49	ESIVRFPNITNLCPF	HLA-DRB3*02:02	0.009	14	324	338
50	NLCPFGEVFNATRFA	HLA-DQA1*05:01/DQB1*02:01	0.01	14	334	348
50	NLCPFGEVFNATRFA	HLA-DQA1*05:01/DQB1*02:01	0.01	14	334	348
51	GEVFNATRFASVYAW	HLA-DRB1*11:01	0.008	14	339	353
51	GEVFNATRFASVYAW	HLA-DRB1*11:01	0.008	14	339	353
52	ATRFASVYAWNRKRI	HLA-DPA1*01:03/DPB1*04:01	0	14	344	358
52	ATRFASVYAWNRKRI	HLA-DPA1*01:03/DPB1*04:01	0	14	344	358
53	RFASVYAWNRKRISN	HLA-DQA1*01:01/DQB1*05:01	0.009	14	346	360
53	RFASVYAWNRKRISN	HLA-DQA1*01:01/DQB1*05:01	0.009	14	346	360
54	YAWNRKRISNCVADY	HLA-DRB1*09:01	0.01	14	351	365
54	YAWNRKRISNCVADY	HLA-DQA1*01:02/DQB1*06:02	0.001	14	351	365
54	YAWNRKRISNCVADY	HLA-DRB1*09:01	0.01	14	351	365
54	YAWNRKRISNCVADY	HLA-DQA1*01:02/DQB1*06:02	0.001	14	351	365
55	FSTFKCYGVSPTKLN	HLA-DQA1*05:01/DQB1*03:01	0.003	14	374	388
55	FSTFKCYGVSPTKLN	HLA-DRB1*09:01	0.002	14	374	388
55	FSTFKCYGVSPTKLN	HLA-DQA1*05:01/DQB1*03:01	0.003	14	374	388
55	FSTFKCYGVSPTKLN	HLA-DRB1*09:01	0.002	14	374	388
56	ADSFVIRGDEVRQIA	HLA-DRB3*02:02	0.003	14	397	411
56	ADSFVIRGDEVRQIA	HLA-DPA1*02:01/DPB1*01:01	0.002	14	397	411
56	ADSFVIRGDEVRQIA	HLA-DRB3*02:02	0.003	14	397	411
56	ADSFVIRGDEVRQIA	HLA-DPA1*02:01/DPB1*01:01	0.002	14	397	411
57	DSFVIRGDEVRQIAP	HLA-DPA1*01:03/DPB1*02:01	0.008	14	398	412
57	DSFVIRGDEVRQIAP	HLA-DPA1*01:03/DPB1*02:01	0.008	14	398	412
58	IRGDEVRQIAPGQTG	HLA-DQA1*01:02/DQB1*06:02	0.006	14	402	416
58	IRGDEVRQIAPGQTG	HLA-DQA1*01:02/DQB1*06:02	0.006	14	402	416
59	GDEVRQIAPGQTGKI	HLA-DRB1*01:01	0	14	404	418
59	GDEVRQIAPGQTGKI	HLA-DRB1*04:01	0.006	14	404	418
59	GDEVRQIAPGQTGKI	HLA-DRB1*09:01	0.01	14	404	418
59	GDEVRQIAPGQTGKI	HLA-DRB1*01:01	0	14	404	418
59	GDEVRQIAPGQTGKI	HLA-DRB1*04:01	0.006	14	404	418
59	GDEVRQIAPGQTGKI	HLA-DRB1*09:01	0.01	14	404	418
60	DEVRQIAPGQTGKIA	HLA-DPA1*03:01/DPB1*04:02	0.006	14	405	419

60	DEVRQIAPGQTGKIA	HLA-DPA1*03:01/DPB1*04:02	0.006	14	405	419
61	VRQIAPGQTGKIADY	HLA-DRB1*12:01	0.01	14	407	421
61	VRQIAPGQTGKIADY	HLA-DRB1*12:01	0.01	14	407	421
62	QIAPGQTGKIADYNY	HLA-DQA1*05:01/DQB1*02:01	0.006	14	409	423
62	QIAPGQTGKIADYNY	HLA-DQA1*05:01/DQB1*02:01	0.006	14	409	423
63	QTGKIADYNYKLPDD	HLA-DRB1*08:02	0.002	14	414	428
63	QTGKIADYNYKLPDD	HLA-DRB1*08:02	0.002	14	414	428
64	FTGCVIAWNSNNLDS	HLA-DRB3*02:02	0.001	14	429	443
64	FTGCVIAWNSNNLDS	HLA-DRB3*02:02	0.001	14	429	443
65	TGCVIAWNSNNLDSK	HLA-DRB1*01:01	0.005	14	430	444
65	TGCVIAWNSNNLDSK	HLA-DRB4*01:01	0.001	14	430	444
65	TGCVIAWNSNNLDSK	HLA-DRB5*01:01	0.005	14	430	444
65	TGCVIAWNSNNLDSK	HLA-DRB1*04:05	0.005	14	430	444
65	TGCVIAWNSNNLDSK	HLA-DRB1*08:02	0.001	14	430	444
65	TGCVIAWNSNNLDSK	HLA-DRB1*13:02	0.008	14	430	444
65	TGCVIAWNSNNLDSK	HLA-DRB1*15:01	0.002	14	430	444
65	TGCVIAWNSNNLDSK	HLA-DRB1*01:01	0.005	14	430	444
65	TGCVIAWNSNNLDSK	HLA-DRB4*01:01	0.001	14	430	444
65	TGCVIAWNSNNLDSK	HLA-DRB5*01:01	0.005	14	430	444
65	TGCVIAWNSNNLDSK	HLA-DRB1*04:05	0.005	14	430	444
65	TGCVIAWNSNNLDSK	HLA-DRB1*08:02	0.001	14	430	444
65	TGCVIAWNSNNLDSK	HLA-DRB1*13:02	0.008	14	430	444
65	TGCVIAWNSNNLDSK	HLA-DRB1*15:01	0.002	14	430	444
66	GCVIAWNSNNLDSKV	HLA-DRB1*03:01	0.002	14	431	445
66	GCVIAWNSNNLDSKV	HLA-DRB1*04:01	0.008	14	431	445
66	GCVIAWNSNNLDSKV	HLA-DRB1*03:01	0.002	14	431	445
66	GCVIAWNSNNLDSKV	HLA-DRB1*04:01	0.008	14	431	445
67	CVIAWNSNNLDSKVG	HLA-DRB3*01:01	0.008	14	432	446
67	CVIAWNSNNLDSKVG	HLA-DRB3*01:01	0.008	14	432	446
68	LDSKVGGNYNYLYRL	HLA-DQA1*05:01/DQB1*02:01	0.003	14	441	455
68	LDSKVGGNYNYLYRL	HLA-DQA1*05:01/DQB1*02:01	0.003	14	441	455
69	KVGGNYNYLYRLFRK	HLA-DPA1*01:03/DPB1*02:01	0	14	444	458
69	KVGGNYNYLYRLFRK	HLA-DPA1*01:03/DPB1*02:01	0	14	444	458
70	SNLKPFERDISTEIY	HLA-DQA1*05:01/DQB1*02:01	0.003	14	459	473
70	SNLKPFERDISTEIY	HLA-DQA1*05:01/DQB1*02:01	0.003	14	459	473
71	KPFERDISTEIYQAG	HLA-DPA1*01:03/DPB1*04:01	0.007	14	462	476
71	KPFERDISTEIYQAG	HLA-DPA1*01:03/DPB1*04:01	0.007	14	462	476
72	TEIYQAGSTPCNGVE	HLA-DRB1*01:01	0.001	14	470	484
72	TEIYQAGSTPCNGVE	HLA-DRB1*04:01	0.001	14	470	484
72	TEIYQAGSTPCNGVE	HLA-DRB5*01:01	0.006	14	470	484
72	TEIYQAGSTPCNGVE	HLA-DRB1*13:02	0.004	14	470	484
72	TEIYQAGSTPCNGVE	HLA-DRB1*01:01	0.001	14	470	484
72	TEIYQAGSTPCNGVE	HLA-DRB1*04:01	0.001	14	470	484
72	TEIYQAGSTPCNGVE	HLA-DRB5*01:01	0.006	14	470	484
72	TEIYQAGSTPCNGVE	HLA-DRB1*13:02	0.004	14	470	484
73	TPCNGVEGFNCYFPL	HLA-DQA1*01:02/DQB1*06:02	0.005	14	478	492
73	TPCNGVEGFNCYFPL	HLA-DQA1*01:02/DQB1*06:02	0.005	14	478	492
74	PYRVVVLSFELLHAP	HLA-DQA1*01:01/DQB1*05:01	0.005	14	507	521
74	PYRVVVLSFELLHAP	HLA-DPA1*02:01/DPB1*01:01	0.001	14	507	521
74	PYRVVVLSFELLHAP	HLA-DQA1*01:01/DQB1*05:01	0.005	14	507	521

74	PYRVVVLSFELLHAP	HLA-DPA1*02:01/DPB1*01:01	0.001	14	507	521
75	VLSFELLHAPATVCG	HLA-DRB5*01:01	0.002	14	512	526
75	VLSFELLHAPATVCG	HLA-DRB5*01:01	0.002	14	512	526
76	KNKCVNFNFNGLTGT	HLA-DRB1*15:01	0.006	14	535	549
76	KNKCVNFNFNGLTGT	HLA-DRB1*15:01	0.006	14	535	549
77	NKCVNFNFNGLTGTG	HLA-DRB1*09:01	0.001	14	536	550
77	NKCVNFNFNGLTGTG	HLA-DRB1*09:01	0.001	14	536	550
78	KCVNFNFNGLTGTGV	HLA-DRB3*02:02	0.006	14	537	551
78	KCVNFNFNGLTGTGV	HLA-DRB3*02:02	0.006	14	537	551
79	GTGVLTESNKKFLPF	HLA-DRB1*13:02	0.005	14	548	562
79	GTGVLTESNKKFLPF	HLA-DRB1*13:02	0.005	14	548	562
80	QFGRDIADTTDAVRD	HLA-DPA1*02:01/DPB1*05:01	0.009	14	564	578
80	QFGRDIADTTDAVRD	HLA-DPA1*02:01/DPB1*05:01	0.009	14	564	578
81	GRDIADTTDAVRDPQ	HLA-DPA1*01:03/DPB1*04:01	0.004	14	566	580
81	GRDIADTTDAVRDPQ	HLA-DPA1*02:01/DPB1*01:01	0.001	14	566	580
81	GRDIADTTDAVRDPQ	HLA-DPA1*01:03/DPB1*04:01	0.004	14	566	580
81	GRDIADTTDAVRDPQ	HLA-DPA1*02:01/DPB1*01:01	0.001	14	566	580
82	RDPQTLEILDITPCS	HLA-DPA1*03:01/DPB1*04:02	0.003	14	577	591
82	RDPQTLEILDITPCS	HLA-DPA1*03:01/DPB1*04:02	0.003	14	577	591
83	GGVSVITPGTNTSNQ	HLA-DQA1*05:01/DQB1*02:01	0.006	14	593	607
83	GGVSVITPGTNTSNQ	HLA-DPA1*02:01/DPB1*05:01	0.005	14	593	607
83	GGVSVITPGTNTSNQ	HLA-DQA1*05:01/DQB1*02:01	0.006	14	593	607
83	GGVSVITPGTNTSNQ	HLA-DPA1*02:01/DPB1*05:01	0.005	14	593	607
84	GTNTSNQVAVLYQDV	HLA-DQA1*01:02/DQB1*06:02	0.007	14	601	615
84	GTNTSNQVAVLYQDV	HLA-DQA1*01:02/DQB1*06:02	0.007	14	601	615
85	QVAVLYQDVNCTEVP	HLA-DRB1*04:01	0.007	14	607	621
85	QVAVLYQDVNCTEVP	HLA-DQA1*05:01/DQB1*03:01	0.004	14	607	621
85	QVAVLYQDVNCTEVP	HLA-DRB1*04:01	0.007	14	607	621
85	QVAVLYQDVNCTEVP	HLA-DQA1*05:01/DQB1*03:01	0.004	14	607	621
86	PTWRVYSTGSNVFQT	HLA-DRB1*04:05	0.007	14	631	645
86	PTWRVYSTGSNVFQT	HLA-DRB1*04:05	0.007	14	631	645
87	GCLIGAEHVNNSYEC	HLA-DRB1*04:01	0.002	14	648	662
87	GCLIGAEHVNNSYEC	HLA-DRB1*04:01	0.002	14	648	662
88	ECDIPIGAGICASYQ	HLA-DRB1*01:01	0.005	14	661	675
88	ECDIPIGAGICASYQ	HLA-DRB4*01:01	0.006	14	661	675
88	ECDIPIGAGICASYQ	HLA-DRB1*01:01	0.005	14	661	675
88	ECDIPIGAGICASYQ	HLA-DRB4*01:01	0.006	14	661	675
89	ICASYQTQTNSPRRA	HLA-DRB1*04:01	0.006	14	670	684
89	ICASYQTQTNSPRRA	HLA-DRB1*04:01	0.006	14	670	684
90	CASYQTQTNSPRRAR	HLA-DRB5*01:01	0.003	14	671	685
90	CASYQTQTNSPRRAR	HLA-DRB1*11:01	0.005	14	671	685
90	CASYQTQTNSPRRAR	HLA-DRB1*13:02	0.001	14	671	685
90	CASYQTQTNSPRRAR	HLA-DRB3*01:01	0.001	14	671	685
90	CASYQTQTNSPRRAR	HLA-DRB5*01:01	0.003	14	671	685
90	CASYQTQTNSPRRAR	HLA-DRB1*11:01	0.005	14	671	685
90	CASYQTQTNSPRRAR	HLA-DRB1*13:02	0.001	14	671	685
90	CASYQTQTNSPRRAR	HLA-DRB3*01:01	0.001	14	671	685
91	ASYQTQTNSPRRARS	HLA-DPA1*01:03/DPB1*02:01	0.002	14	672	686
91	ASYQTQTNSPRRARS	HLA-DRB3*02:02	0.008	14	672	686
91	ASYQTQTNSPRRARS	HLA-DPA1*01:03/DPB1*04:01	0.001	14	672	686

91	ASYQTQTNSPRRARS	HLA-DPA1*03:01/DPB1*04:02	0.003	14	672	686
91	ASYQTQTNSPRRARS	HLA-DPA1*01:03/DPB1*02:01	0.002	14	672	686
91	ASYQTQTNSPRRARS	HLA-DRB3*02:02	0.008	14	672	686
91	ASYQTQTNSPRRARS	HLA-DPA1*01:03/DPB1*04:01	0.001	14	672	686
91	ASYQTQTNSPRRARS	HLA-DPA1*03:01/DPB1*04:02	0.003	14	672	686
92	SYQTQTNSPRRARSV	HLA-DRB1*03:01	0.001	14	673	687
92	SYQTQTNSPRRARSV	HLA-DRB1*03:01	0.001	14	673	687
93	SPRRARSVASQSIIA	HLA-DQA1*01:01/DQB1*05:01	0.004	14	680	694
93	SPRRARSVASQSIIA	HLA-DQA1*01:01/DQB1*05:01	0.004	14	680	694
94	SQSIIAYTMSLGAEN	HLA-DRB4*01:01	0.009	14	689	703
94	SQSIIAYTMSLGAEN	HLA-DRB1*09:01	0.002	14	689	703
94	SQSIIAYTMSLGAEN	HLA-DRB4*01:01	0.009	14	689	703
94	SQSIIAYTMSLGAEN	HLA-DRB1*09:01	0.002	14	689	703
95	ENSVAYSNNSIAIPT	HLA-DRB1*15:01	0	14	702	716
95	ENSVAYSNNSIAIPT	HLA-DRB3*01:01	0.009	14	702	716
95	ENSVAYSNNSIAIPT	HLA-DRB1*15:01	0	14	702	716
95	ENSVAYSNNSIAIPT	HLA-DRB3*01:01	0.009	14	702	716
96	GDSTECSNLLLQYGS	HLA-DPA1*03:01/DPB1*04:02	0.008	14	744	758
96	GDSTECSNLLLQYGS	HLA-DPA1*03:01/DPB1*04:02	0.008	14	744	758
97	ECSNLLLQYGSFCTQ	HLA-DRB1*11:01	0.009	14	748	762
97	ECSNLLLQYGSFCTQ	HLA-DRB1*11:01	0.009	14	748	762
98	QYGSFCTQLNRALTG	HLA-DRB4*01:01	0.008	14	755	769
98	QYGSFCTQLNRALTG	HLA-DRB1*04:05	0.005	14	755	769
98	QYGSFCTQLNRALTG	HLA-DRB1*07:01	0.005	14	755	769
98	QYGSFCTQLNRALTG	HLA-DRB4*01:01	0.008	14	755	769
98	QYGSFCTQLNRALTG	HLA-DRB1*04:05	0.005	14	755	769
98	QYGSFCTQLNRALTG	HLA-DRB1*07:01	0.005	14	755	769
99	GSFCTQLNRALTGIA	HLA-DRB3*02:02	0.007	14	757	771
99	GSFCTQLNRALTGIA	HLA-DPA1*01:03/DPB1*04:01	0.01	14	757	771
99	GSFCTQLNRALTGIA	HLA-DPA1*02:01/DPB1*01:01	0.003	14	757	771
99	GSFCTQLNRALTGIA	HLA-DRB3*02:02	0.007	14	757	771
99	GSFCTQLNRALTGIA	HLA-DPA1*01:03/DPB1*04:01	0.01	14	757	771
99	GSFCTQLNRALTGIA	HLA-DPA1*02:01/DPB1*01:01	0.003	14	757	771
100	TQLNRALTGIAVEQD	HLA-DRB1*12:01	0.002	14	761	775
100	TQLNRALTGIAVEQD	HLA-DRB1*12:01	0.002	14	761	775
101	QLNRALTGIAVEQDK	HLA-DQA1*01:02/DQB1*06:02	0.002	14	762	776
101	QLNRALTGIAVEQDK	HLA-DQA1*01:02/DQB1*06:02	0.002	14	762	776
102	QDKNTQEVFAQVKQI	HLA-DPA1*01:03/DPB1*04:01	0.001	14	774	788
102	QDKNTQEVFAQVKQI	HLA-DPA1*01:03/DPB1*04:01	0.001	14	774	788
103	QEVFAQVKQIYKTPP	HLA-DRB1*12:01	0.007	14	779	793
103	QEVFAQVKQIYKTPP	HLA-DRB1*12:01	0.007	14	779	793
104	DFGGFNFSQILPDPS	HLA-DRB1*07:01	0.007	14	796	810
104	DFGGFNFSQILPDPS	HLA-DRB1*07:01	0.007	14	796	810
105	FGGFNFSQILPDPSK	HLA-DRB4*01:01	0.001	14	797	811
105	FGGFNFSQILPDPSK	HLA-DRB4*01:01	0.001	14	797	811
106	GFNFSQILPDPSKPS	HLA-DRB1*04:01	0.005	14	799	813
106	GFNFSQILPDPSKPS	HLA-DRB1*04:01	0.005	14	799	813
107	FSQILPDPSKPSKRS	HLA-DRB1*01:01	0.005	14	802	816
107	FSQILPDPSKPSKRS	HLA-DRB1*01:01	0.005	14	802	816
108	PSKPSKRSFIEDLLF	HLA-DQA1*05:01/DQB1*02:01	0.009	14	809	823

108	PSKPSKRSFIEDLLF	HLA-DQA1*05:01/DQB1*02:01	0.009	14	809	823
109	SKRSFIEDLLFNKVT	HLA-DPA1*03:01/DPB1*04:02	0.003	14	813	827
109	SKRSFIEDLLFNKVT	HLA-DPA1*03:01/DPB1*04:02	0.003	14	813	827
110	RSFIEDLLFNKVTLA	HLA-DRB1*15:01	0.009	14	815	829
110	RSFIEDLLFNKVTLA	HLA-DRB1*15:01	0.009	14	815	829
111	IEDLLFNKVTLADAG	HLA-DRB1*04:01	0.009	14	818	832
111	IEDLLFNKVTLADAG	HLA-DRB1*04:01	0.009	14	818	832
112	AQKFNGLTVLPPLLT	HLA-DRB3*01:01	0.001	14	852	866
112	AQKFNGLTVLPPLLT	HLA-DRB3*01:01	0.001	14	852	866
113	QKFNGLTVLPPLLTD	HLA-DRB1*07:01	0.003	14	853	867
113	QKFNGLTVLPPLLTD	HLA-DRB1*07:01	0.003	14	853	867
114	KFNGLTVLPPLLTDE	HLA-DRB1*08:02	0.007	14	854	868
114	KFNGLTVLPPLLTDE	HLA-DQA1*01:02/DQB1*06:02	0.01	14	854	868
114	KFNGLTVLPPLLTDE	HLA-DRB1*08:02	0.007	14	854	868
114	KFNGLTVLPPLLTDE	HLA-DQA1*01:02/DQB1*06:02	0.01	14	854	868
115	TDEMIAQYTSALLAG	HLA-DRB5*01:01	0.004	14	866	880
115	TDEMIAQYTSALLAG	HLA-DRB5*01:01	0.004	14	866	880
116	DEMIAQYTSALLAGT	HLA-DRB1*01:01	0.005	14	867	881
116	DEMIAQYTSALLAGT	HLA-DRB1*09:01	0.002	14	867	881
116	DEMIAQYTSALLAGT	HLA-DRB1*13:02	0.005	14	867	881
116	DEMIAQYTSALLAGT	HLA-DRB1*01:01	0.005	14	867	881
116	DEMIAQYTSALLAGT	HLA-DRB1*09:01	0.002	14	867	881
116	DEMIAQYTSALLAGT	HLA-DRB1*13:02	0.005	14	867	881
117	AGTITSGWTFGAGAA	HLA-DRB4*01:01	0.005	14	879	893
117	AGTITSGWTFGAGAA	HLA-DRB4*01:01	0.005	14	879	893
118	GIGVTQNVLYENQKL	HLA-DQA1*01:01/DQB1*05:01	0.006	14	908	922
118	GIGVTQNVLYENQKL	HLA-DPA1*03:01/DPB1*04:02	0.007	14	908	922
118	GIGVTQNVLYENQKL	HLA-DQA1*01:01/DQB1*05:01	0.006	14	908	922
118	GIGVTQNVLYENQKL	HLA-DPA1*03:01/DPB1*04:02	0.007	14	908	922
119	SAIGKIQDSLSSTAS	HLA-DQA1*01:01/DQB1*05:01	0.004	14	929	943
119	SAIGKIQDSLSSTAS	HLA-DQA1*01:01/DQB1*05:01	0.004	14	929	943
120	AIGKIQDSLSSTASA	HLA-DRB1*08:02	0.006	14	930	944
120	AIGKIQDSLSSTASA	HLA-DRB1*08:02	0.006	14	930	944
121	IQDSLSSTASALGKL	HLA-DQA1*05:01/DQB1*03:01	0.004	14	934	948
121	IQDSLSSTASALGKL	HLA-DRB3*02:02	0.01	14	934	948
121	IQDSLSSTASALGKL	HLA-DQA1*05:01/DQB1*03:01	0.004	14	934	948
121	IQDSLSSTASALGKL	HLA-DRB3*02:02	0.01	14	934	948
122	SSNFGAISSVLNDIL	HLA-DQA1*05:01/DQB1*02:01	0.01	14	967	981
122	SSNFGAISSVLNDIL	HLA-DQA1*05:01/DQB1*02:01	0.01	14	967	981
123	EAEVQIDRLITGRLQ	HLA-DRB1*11:01	0.002	14	988	1002
123	EAEVQIDRLITGRLQ	HLA-DRB1*11:01	0.002	14	988	1002
124	QSLQTYVTQQLIRAA	HLA-DRB3*01:01	0.006	14	1002	1016
124	QSLQTYVTQQLIRAA	HLA-DRB3*01:01	0.006	14	1002	1016
125	LQTYVTQQLIRAAEI	HLA-DRB4*01:01	0.008	14	1004	1018
125	LQTYVTQQLIRAAEI	HLA-DRB4*01:01	0.008	14	1004	1018
126	QQLIRAAEIRASANL	HLA-DQA1*01:02/DQB1*06:02	0.004	14	1010	1024
126	QQLIRAAEIRASANL	HLA-DQA1*01:02/DQB1*06:02	0.004	14	1010	1024
127	SECVLGQSKRVDFCG	HLA-DRB4*01:01	0.009	14	1030	1044
127	SECVLGQSKRVDFCG	HLA-DRB4*01:01	0.009	14	1030	1044
128	FCGKGYHLMSFPQSA	HLA-DQA1*01:01/DQB1*05:01	0.006	14	1042	1056

128	FCGKGYHLMSFPQSA	HLA-DRB3*02:02	0.009	14	1042	1056
128	FCGKGYHLMSFPQSA	HLA-DQA1*01:01/DQB1*05:01	0.006	14	1042	1056
128	FCGKGYHLMSFPQSA	HLA-DRB3*02:02	0.009	14	1042	1056
129	GKGYHLMSFPQSAPH	HLA-DRB1*04:01	0.007	14	1044	1058
129	GKGYHLMSFPQSAPH	HLA-DPA1*01:03/DPB1*02:01	0.009	14	1044	1058
129	GKGYHLMSFPQSAPH	HLA-DRB1*04:01	0.007	14	1044	1058
129	GKGYHLMSFPQSAPH	HLA-DPA1*01:03/DPB1*02:01	0.009	14	1044	1058
130	KGYHLMSFPQSAPHG	HLA-DRB1*03:01	0.004	14	1045	1059
130	KGYHLMSFPQSAPHG	HLA-DRB1*08:02	0.001	14	1045	1059
130	KGYHLMSFPQSAPHG	HLA-DRB1*03:01	0.004	14	1045	1059
130	KGYHLMSFPQSAPHG	HLA-DRB1*08:02	0.001	14	1045	1059
131	GYHLMSFPQSAPHGV	HLA-DQA1*05:01/DQB1*03:01	0.007	14	1046	1060
131	GYHLMSFPQSAPHGV	HLA-DQA1*05:01/DQB1*03:01	0.007	14	1046	1060
132	YHLMSFPQSAPHGVV	HLA-DRB1*09:01	0.007	14	1047	1061
132	YHLMSFPQSAPHGVV	HLA-DRB1*12:01	0.002	14	1047	1061
132	YHLMSFPQSAPHGVV	HLA-DRB1*09:01	0.007	14	1047	1061
132	YHLMSFPQSAPHGVV	HLA-DRB1*12:01	0.002	14	1047	1061
133	PHGVVFLHVTYVPAQ	HLA-DRB1*11:01	0.004	14	1057	1071
133	PHGVVFLHVTYVPAQ	HLA-DRB1*11:01	0.004	14	1057	1071
134	HGVVFLHVTYVPAQE	HLA-DRB3*01:01	0.009	14	1058	1072
134	HGVVFLHVTYVPAQE	HLA-DRB3*01:01	0.009	14	1058	1072
135	PAQEKNFTTAPAICH	HLA-DRB1*13:02	0.005	14	1069	1083
135	PAQEKNFTTAPAICH	HLA-DRB1*13:02	0.005	14	1069	1083
136	AQEKNFTTAPAICHD	HLA-DQA1*05:01/DQB1*03:01	0.005	14	1070	1084
136	AQEKNFTTAPAICHD	HLA-DRB1*08:02	0.003	14	1070	1084
136	AQEKNFTTAPAICHD	HLA-DQA1*05:01/DQB1*03:01	0.005	14	1070	1084
136	AQEKNFTTAPAICHD	HLA-DRB1*08:02	0.003	14	1070	1084
137	QEKNFTTAPAICHDG	HLA-DRB5*01:01	0.002	14	1071	1085
137	QEKNFTTAPAICHDG	HLA-DRB1*04:05	0.007	14	1071	1085
137	QEKNFTTAPAICHDG	HLA-DQA1*01:02/DQB1*06:02	0.01	14	1071	1085
137	QEKNFTTAPAICHDG	HLA-DRB3*02:02	0.002	14	1071	1085
137	QEKNFTTAPAICHDG	HLA-DRB5*01:01	0.002	14	1071	1085
137	QEKNFTTAPAICHDG	HLA-DRB1*04:05	0.007	14	1071	1085
137	QEKNFTTAPAICHDG	HLA-DQA1*01:02/DQB1*06:02	0.01	14	1071	1085
137	QEKNFTTAPAICHDG	HLA-DRB3*02:02	0.002	14	1071	1085
138	EKNFTTAPAICHDGK	HLA-DRB1*01:01	0.01	14	1072	1086
138	EKNFTTAPAICHDGK	HLA-DRB1*01:01	0.01	14	1072	1086
139	CHDGKAHFPREGVFV	HLA-DRB1*12:01	0.003	14	1082	1096
139	CHDGKAHFPREGVFV	HLA-DRB1*12:01	0.003	14	1082	1096
140	REGVFVSNGTHWFVT	HLA-DRB5*01:01	0.006	14	1091	1105
140	REGVFVSNGTHWFVT	HLA-DRB5*01:01	0.006	14	1091	1105
141	FVSNGTHWFVTQRNF	HLA-DQA1*05:01/DQB1*02:01	0.005	14	1095	1109
141	FVSNGTHWFVTQRNF	HLA-DQA1*05:01/DQB1*02:01	0.005	14	1095	1109
142	PQIITTDNTFVSGNC	HLA-DRB3*01:01	0.01	14	1112	1126
142	PQIITTDNTFVSGNC	HLA-DRB3*01:01	0.01	14	1112	1126
143	IITTDNTFVSGNCDV	HLA-DQA1*05:01/DQB1*03:01	0.004	14	1114	1128
143	IITTDNTFVSGNCDV	HLA-DQA1*05:01/DQB1*03:01	0.004	14	1114	1128
144	NCDVVIGIVNNTVYD	HLA-DRB1*01:01	0.001	14	1125	1139
144	NCDVVIGIVNNTVYD	HLA-DRB1*04:01	0.002	14	1125	1139
144	NCDVVIGIVNNTVYD	HLA-DQA1*05:01/DQB1*02:01	0.003	14	1125	1139

144	NCDVVIGIVNNTVYD	HLA-DPA1*02:01/DPB1*01:01	0.004	14	1125	1139
144	NCDVVIGIVNNTVYD	HLA-DRB1*01:01	0.001	14	1125	1139
144	NCDVVIGIVNNTVYD	HLA-DRB1*04:01	0.002	14	1125	1139
144	NCDVVIGIVNNTVYD	HLA-DQA1*05:01/DQB1*02:01	0.003	14	1125	1139
144	NCDVVIGIVNNTVYD	HLA-DPA1*02:01/DPB1*01:01	0.004	14	1125	1139
145	KEELDKYFKNHTSPD	HLA-DRB1*15:01	0	14	1149	1163
145	KEELDKYFKNHTSPD	HLA-DPA1*01:03/DPB1*04:01	0.01	14	1149	1163
145	KEELDKYFKNHTSPD	HLA-DRB1*15:01	0	14	1149	1163
145	KEELDKYFKNHTSPD	HLA-DPA1*01:03/DPB1*04:01	0.01	14	1149	1163
146	EELDKYFKNHTSPDV	HLA-DRB1*04:05	0.005	14	1150	1164
146	EELDKYFKNHTSPDV	HLA-DRB1*11:01	0.002	14	1150	1164
146	EELDKYFKNHTSPDV	HLA-DRB1*04:05	0.005	14	1150	1164
146	EELDKYFKNHTSPDV	HLA-DRB1*11:01	0.002	14	1150	1164
147	ELDKYFKNHTSPDVD	HLA-DRB1*08:02	0.001	14	1151	1165
147	ELDKYFKNHTSPDVD	HLA-DRB1*08:02	0.001	14	1151	1165
148	LDKYFKNHTSPDVDL	HLA-DRB1*01:01	0.003	14	1152	1166
148	LDKYFKNHTSPDVDL	HLA-DRB1*04:01	0.003	14	1152	1166
148	LDKYFKNHTSPDVDL	HLA-DRB4*01:01	0.01	14	1152	1166
148	LDKYFKNHTSPDVDL	HLA-DRB1*07:01	0.001	14	1152	1166
148	LDKYFKNHTSPDVDL	HLA-DRB1*09:01	0	14	1152	1166
148	LDKYFKNHTSPDVDL	HLA-DRB1*01:01	0.003	14	1152	1166
148	LDKYFKNHTSPDVDL	HLA-DRB1*04:01	0.003	14	1152	1166
148	LDKYFKNHTSPDVDL	HLA-DRB4*01:01	0.01	14	1152	1166
148	LDKYFKNHTSPDVDL	HLA-DRB1*07:01	0.001	14	1152	1166
148	LDKYFKNHTSPDVDL	HLA-DRB1*09:01	0	14	1152	1166
149	DKYFKNHTSPDVDLG	HLA-DRB5*01:01	0.001	14	1153	1167
149	DKYFKNHTSPDVDLG	HLA-DQA1*05:01/DQB1*02:01	0.006	14	1153	1167
149	DKYFKNHTSPDVDLG	HLA-DRB3*02:02	0.002	14	1153	1167
149	DKYFKNHTSPDVDLG	HLA-DRB5*01:01	0.001	14	1153	1167
149	DKYFKNHTSPDVDLG	HLA-DQA1*05:01/DQB1*02:01	0.006	14	1153	1167
149	DKYFKNHTSPDVDLG	HLA-DRB3*02:02	0.002	14	1153	1167
150	VDLGDISGINASVVN	HLA-DQA1*05:01/DQB1*03:01	0.004	14	1164	1178
150	VDLGDISGINASVVN	HLA-DQA1*05:01/DQB1*03:01	0.004	14	1164	1178
151	EVAKNLNESLIDLQE	HLA-DQA1*01:01/DQB1*05:01	0.004	14	1188	1202
151	EVAKNLNESLIDLQE	HLA-DQA1*01:01/DQB1*05:01	0.004	14	1188	1202
152	WPWYIWLGFIAGLIA	HLA-DRB1*04:05	0.004	14	1212	1226
152	WPWYIWLGFIAGLIA	HLA-DRB1*04:05	0.004	14	1212	1226
153	PWYIWLGFIAGLIAI	HLA-DPA1*01:03/DPB1*04:01	0.009	14	1213	1227
153	PWYIWLGFIAGLIAI	HLA-DPA1*01:03/DPB1*04:01	0.009	14	1213	1227
154	WLGFIAGLIAIVMVT	HLA-DRB1*07:01	0.008	14	1217	1231
154	WLGFIAGLIAIVMVT	HLA-DRB1*07:01	0.008	14	1217	1231
155	TSCCSCLKGCCSCGS	HLA-DQA1*05:01/DQB1*03:01	0.005	14	1238	1252
155	TSCCSCLKGCCSCGS	HLA-DQA1*05:01/DQB1*03:01	0.005	14	1238	1252
156	GSCCKFDEDDSEPVL	HLA-DQA1*01:01/DQB1*05:01	0.007	14	1251	1265
156	GSCCKFDEDDSEPVL	HLA-DQA1*01:01/DQB1*05:01	0.007	14	1251	1265
157	CCKFDEDDSEPVLKG	HLA-DRB1*01:01	0.003	14	1253	1267
157	CCKFDEDDSEPVLKG	HLA-DRB1*04:01	0.002	14	1253	1267
157	CCKFDEDDSEPVLKG	HLA-DRB1*13:02	0.001	14	1253	1267
157	CCKFDEDDSEPVLKG	HLA-DPA1*03:01/DPB1*04:02	0.003	14	1253	1267
157	CCKFDEDDSEPVLKG	HLA-DRB1*01:01	0.003	14	1253	1267

157	CCKFDEDDSEPVLKG	HLA-DRB1*04:01	0.002	14	1253	1267
157	CCKFDEDDSEPVLKG	HLA-DRB1*13:02	0.001	14	1253	1267
157	CCKFDEDDSEPVLKG	HLA-DPA1*03:01/DPB1*04:02	0.003	14	1253	1267
158	CKFDEDDSEPVLKGV	HLA-DRB1*03:01	0.004	14	1254	1268
158	CKFDEDDSEPVLKGV	HLA-DRB3*01:01	0.002	14	1254	1268
158	CKFDEDDSEPVLKGV	HLA-DRB1*03:01	0.004	14	1254	1268
158	CKFDEDDSEPVLKGV	HLA-DRB3*01:01	0.002	14	1254	1268

Table S5. IEDB mapped B cell epitopes for S protein.

IEDB ID	Epitope	Starting position	Ending position
462	AATKMSECVLGQSKRVD	1024	1041
2092	AISSVLNDILSRLDKVE	971	988
3176	AMQMAYRF	898	906
4129	ARDLICAQKFNGLTVLP	845	862
6476	CKFDEDDSEPVLKGVKLHYT	1253	1273
7868	DDSEPVLKGVKLHYT	1258	1273
9007	DKYFKNHTSPDVDLGD	1152	1168
9094	DLGDISGINASVVNIQK	1164	1181
10113	DSFKEELDKYFKNHTSPDVDLGDISGINASVV	1145	1177
10778	DVVNQNAQALNTLVKQL	949	966
11038	EAEVQIDRLITGRLQSL	987	1004
11740	EELDKYFKNHTSPDVDL	1149	1166
12426	EIDRLNEVAKNLNESLIDLQELGKYEQY	1181	1209
14626	EVAKNLNESLIDLQELG	1187	1204
15972	FGEVFNAT	337	345
16183	FIEDLLFNKVTLADAGF	816	833
18515	GAALQIPFAMQMAYRFN	890	907
18594	GAGICASY	666	674
22321	GSFCTQLN	756	764
27357	ILSRLDKVEAEVQIDRL	979	996
28512	ISGINASVVNIQKEIDRLNE	1168	1188
28513	ISGINASVVNIQKEIDRLNEVAKNLNESLIDLQELGKYEQYI	1168	1210
29108	ITTDNTFVSGNCDVVIG	1114	1131
30435	KEIDRLNEVAKNLNESL	1180	1197
30987	KGIYQTSN	309	317
32508	KNHTSPDVDLGDISGIN	1156	1173
33032	KQLSSNFGAISSVLNDI	963	980
41177	MAYRFNGIGVTQNVLYE	901	918
47341	PELDSFKEELDKYFKNH	1142	1159
47479	PFAMQMAYRFNGIGVTQ	896	913
50311	QALNTLVKQLSSNFGAI	956	973
51379	QLIRAAEIRASANLAAT	1010	1027
52020	QQFGRD	562	568
53202	RASANLAATKMSECVLG	1018	1035
54599	RLITGRLQSLQTYVTQQ	994	1011
59425	SLQTYVTQQLIRAAEIR	1002	1019
60024	SPDVDLGDISGINAS	1160	1175
67220	TVYDPLQPELDSFKEEL	1135	1152
69513	VLGQSKRVDFCGKGYHL	1032	1049
70719	VRFPNITNLCPFGEVFN	326	343

462413	PLQPE	1139	1144
558417	EIDRLNEVAKNLNESLIDLQELGKYEQY	1181	1209
558455	LYQDVN	610	616
558456	LYQDVNC	610	617
558457	LYQDVNCT	610	618

Table S6. IEDB mapped T cell epitopes for S protein.

IEDB ID	Epitope	MHC restriction	Starting position	Ending position
2801	ALNTLVKQL	HLA-A*02:01	957	966
16156	FIAGLIAIV	H2 class I; HLA-A2; HLA-A*02:01	1219	1228
36724	LITGRLQSL	H2 class I; HLA-A2; HLA-A*02:01	995	1004
44814	NLNESLIDL	HLA-A*02:01	1191	1200
50311	QALNTLVKQLSSNFGAI	HLA-DRB1*04:01	956	973
54680	RLNEVAKNL	H2 class I; HLA-A*02:01	1184	1193
69657	VLNDILSRL	HLA-A*02:01	975	984
70066	VNFNFNGL	H2-Kb; H2-b class I	538	546
71663	VVFLHVTYV	HLA-A*02:01	1059	1068
100048	GAALQIPFAMQMAYRF	HLA-DRA*01:01/DRB1*07:01	890	906
100300	MAYRFNGIGVTQNVLY	HLA-DRB1*04:01	901	917
100428	QLIRAAEIRASANLAATK	HLA-DRB1*04:01	1010	1028

Table S7. Vaxign2 EggNOG Gene Ontology prediction for S protein.

	Gene Ontology (GO)
Biological Process	adhesion of symbiont to host (GO:0044406)
	membrane fusion (GO:0061025)
	viral life cycle (GO:0019058)
	cellular component organization (GO:0016043)
	interaction with host (GO:0051701)
	symbiotic process (GO:0044403)
	viral process (GO:0016032)
	membrane organization (GO:0061024)
	multi-organism process (GO:0051704)
	entry into host (GO:0044409)
	membrane fusion involved in viral entry into host cell (GO:0039663)
	viral entry into host cell (GO:0046718)
	fusion of virus membrane with host plasma membrane (GO:0019064)
	biological_process (GO:0008150)
	entry into host (GO:0051828)
	cellular process (GO:0009987)
	interspecies interaction between organisms (GO:0044419)
	cellular component organization or biogenesis (GO:0071840)
	biological adhesion (GO:0022610)
	virion attachment to host cell (GO:0019062)
	multi-organism membrane fusion (GO:0044800)
	entry into host cell (GO:0030260)
	receptor-mediated virion attachment to host cell (GO:0046813)
	entry into host cell (GO:0051806)
	adhesion of symbiont to host cell (GO:0044650)
	multi-organism membrane organization (GO:0044803)
	multi-organism cellular process (GO:0044764)
Molecular Function	host cell surface binding (GO:0046812)
	host cell surface receptor binding (GO:0046789)
	binding (GO:0005488)
	protein binding (GO:0005515)
	molecular_function (GO:0003674)
	identical protein binding (GO:0042802)
Cellular Component	host cell cytoplasm (GO:0030430)
	host intracellular part (GO:0033646)
	host cell Golgi apparatus (GO:0044177)
	host cell cytoplasm part (GO:0033655)
	host cell (GO:0043657)
	other organism cell (GO:0044216)
	host cellular component (GO:0018995)

host intracellular region (GO:0043656)

other organism part (GO:0044217)

cellular_component (GO:0005575)

host cell part (GO:0033643)

Table S8. Vaxign2 EggNOG Gene Ontology prediction for S protein.

Organism	Protein
Alphacoronavirus BtMs-AlphaCoV/GS2013 (Taxon: 1503290)	A0A0U1UZ37_9ALPC
Avian infectious bronchitis virus (strain Beaudette) (Taxon: 11122)	SPIKE_IBVB
Bat coronavirus 1A (Taxon: 393767)	B1PHJ5_9ALPC
Bat coronavirus Cp/Yunnan2011 (Taxon: 1283333)	R9QTH3_CVHSA
Bat coronavirus HKU9-2 (Taxon: 424368)	A3EXH4_BCHK9
Bat Hp-betacoronavirus/Zhejiang2013 (Taxon: 1541205)	A0A088DJY6_9BETC
Bat SARS CoV Rm1/2004 (Taxon: 347536)	Q0QDX9_CVHSA
Bat SARS-like coronavirus YNLF_31C (Taxon: 1699360)	A0A0K1Z074_CVHSA
Betacoronavirus England 1 (Taxon: 1263720)	SPIKE_CVEMC
Betacoronavirus Erinaceus/VMC/DEU/2012 (Taxon: 1385427)	U5LNM4_9BETC
Betacoronavirus HKU24 (Taxon: 1590370)	A0A0A7UZR7_9BETC
Bottlenose dolphin coronavirus HKU22 (Taxon: 1433215)	V5TFD8_9GAMC
Bovine coronavirus isolate Alpaca (Taxon: 404135)	Q06BD7_9BETC
BtMf-AlphaCoV/GD2012 (Taxon: 1503280)	A0A0U1WHB6_9ALPC
BtMr-AlphaCoV/SAX2011 (Taxon: 1503289)	A0A0U1WHD7_9ALPC
BtNv-AlphaCoV/SC2013 (Taxon: 1503291)	A0A0U1UZD0_9ALPC
BtRf-AlphaCoV/YN2012 (Taxon: 1503293)	A0A0U1WJW4_9ALPC
BtVs-BetaCoV/SC2013 (Taxon: 1495253)	A0A023Y9K3_9BETC
Bulbul coronavirus HKU11-796 (Taxon: 572287)	B6VDW9_9NIDO
Camel alphacoronavirus (Taxon: 1699095)	A0A0U2GRB2_CVH22
Canine coronavirus (Taxon: 11153)	H9TEX4_9ALPC
Common moorhen coronavirus HKU21 (Taxon: 1159902)	H9BR35_9NIDO
European turkey coronavirus 080385d (Taxon: 1763410)	A0A0S2ZWY7_9GAMC
Feline coronavirus (Taxon: 12663)	A0A125R5A5_9ALPC
Feline infectious peritonitis virus (strain 79-1146) (Taxon: 33734)	SPIKE_FIPV
Ferret coronavirus (Taxon: 1264898)	A0A172AZS6_9ALPC
Hipposideros bat coronavirus HKU10 (Taxon: 1241932)	K4JZP8_9ALPC
Human coronavirus 229E (Taxon: 11137)	SPIKE_CVH22
Human coronavirus NL63 (Taxon: 277944)	SPIKE_CVHNL
Human coronavirus OC43 (Taxon: 31631)	Q4VID5_CVHOC
Infectious bronchitis virus NGA/A116E7/2006 (Taxon: 658930)	D0R6S1_9GAMC
Miniopterus bat coronavirus HKU8 (Taxon: 694001)	B1PHK2_9ALPC
Mink coronavirus strain WD1133 (Taxon: 766792)	D9J204_9ALPC
Murine hepatitis virus (Taxon: 11138)	Q9J3E7_9BETC
Murine hepatitis virus strain 2 (Taxon: 76344)	Q77NQ7_CVM2
Murine hepatitis virus strain A59 (Taxon: 11142)	SPIKE_CVMA5
Murine hepatitis virus strain S/3239-17 (Taxon: 1163669)	H9BZX9_9BETC
Night heron coronavirus HKU19 (Taxon: 1159904)	H9BR17_9NIDO
Pipistrellus bat coronavirus HKU5 (Taxon: 694008)	SPIKE_BCHK5
Porcine deltacoronavirus (Taxon: 1586324)	A0A140ESF1_9NIDO

Porcine epidemic diarrhea virus CV777 (Taxon: 229032)	SPIKE_PEDV7
Porcine hemagglutinating encephalomyelitis virus (strain 67N) (Taxon: 230237)	SPIKE_CVP67
Porcine transmissible gastroenteritis coronavirus strain Purdue (Taxon: 11151)	SPIKE_CVPPU
Rabbit coronavirus HKU14 (Taxon: 1160968)	H9AA65_9BETC
Scotophilus bat coronavirus 512 (Taxon: 693999)	SPIKE_BC512
Sparrow coronavirus HKU17 (Taxon: 1159906)	H9BR00_9NIDO
Thrush coronavirus HKU12-600 (Taxon: 572290)	B6VDX8_THCOV
Transmissible gastroenteritis virus (Taxon: 11149)	A0A0Y0RQT7_9ALPC
Tylonycteris bat coronavirus HKU4 (Taxon: 694007)	SPIKE_BCHK4
Wigeon coronavirus HKU20 (Taxon: 1159908)	H9BR25_9NIDO
(Taxon: 227859)	SPIKE_CVHSA
